# Prevalence of cerebral palsy comorbidities in China: a systematic review and meta-analysis

**DOI:** 10.3389/fneur.2023.1233700

**Published:** 2023-09-28

**Authors:** Chao Gong, Xiaopei Liu, Liya Fang, Annan Liu, Beibei Lian, Xunzhong Qi, Shuyue Chen, Huiqing Li, Ming Zhao, Jin Guo, Shaobo Zhou

**Affiliations:** ^1^College of Rehabilitation Medicine, Jiamusi University, Jiamusi, China; ^2^Jiamusi University Affiliated No. 3 Hospital, Jiamusi, China; ^3^Jiamusi University Affiliated No. 1 Hospital, Jiamusi, China; ^4^College of Basic Medicine, Jiamusi University, Jiamusi, China; ^5^College of Public Health, Jiamusi University, Jiamusi, China; ^6^School of Science, Faculty of Engineering and Science, University of Greenwich, Medway Campus Central Avenue, Chatham Maritime, Kent, England

**Keywords:** cerebral palsy, comorbidities, epilepsy, prevalence, meta-analyzes, China

## Abstract

**Objectives:**

This systematic review aimed to comprehensively understand the comorbidity of cerebral palsy (CP) in China.

**Methods:**

We searched through databases in both Chinese and English until December 2022 to gather cross-sectional studies on the comorbidity of CP in China. After two reviewers independently screened the articles, collected the data, and assessed the bias risk, a meta-analysis was conducted using the Stata 17.0 software.

**Results:**

A total of 73 articles were included. Of these, 16 articles reported total comorbidity, with a prevalence of 79.7% (95% CI: 73.8–85.7%); 56 articles reported epilepsy, with a prevalence of 17.9% (95% CI: 15.4–20.4%); 48 articles reported intellectual disability, with a prevalence of 58.0% (95% CI: 51.8–64.3%); 32 articles reported speech disorders, with a prevalence of 48.0% (95% CI: 41.6–54.4%); 41 articles reported hearing disorders, with a prevalence of 17.2% (95% CI: 13.0–21.4%); and 35 articles reported vision disorders, with a prevalence of 23.1% (95% CI: 16.3–29.8%). The topographical type of CP was the primary source of heterogeneity in the prevalence of epilepsy. Diagnostic criteria for CP, clinical type of CP, GMFCS, publishing time, and topographical type of CP were the primary sources of heterogeneity in the prevalence of intellectual disability. Clinical type of CP and topographical type were the primary sources of heterogeneity in the prevalence of speech disorders. Finally, the region was the primary source of heterogeneity in the prevalence of hearing disorders.

**Conclusion:**

The prevalence of comorbidities in CP is high in China. Comorbidities are related to the characteristics, severity, and risk factors of brain insult and have a particular relationship with regional economic development and medical and health levels.

## 1. Introduction

Cerebral palsy (CP) is a joint complex and multifactorial chronic neurological disorder that occurs in childhood due to non-progressive abnormalities in postural control, muscle tone, and motor function caused by permanent damage to the brain during early development (1, 2). However, pure motor deficits are rare in CP, and the diversity of neurological insult is inevitably accompanied by many different levels of functional impairment and disease; examples include sensation, perception, cognition, communication, behaviour, epilepsy, and secondary musculoskeletal problems (2).

According to data from high-income nations, CP is frequently accompanied by different levels of related impairment (3, 4). According to a recent study analyzing data from a Norwegian registry of those with the condition, 95% of children with CP had at least one comorbidity (3). The European CP Monitor states that the most frequent comorbidities are speech disorders, intellectual disability, epilepsy, and vision disorders (4). Comorbidities in children with CP are a severe problem. Due to their motor dysfunction, children with CP have impaired motor function. When comorbidities are present, they can substantially hinder rehabilitation training and decrease the likelihood of the child’s survival and quality of life in the future. Our understanding of the complexity of CP can be enhanced by studying the comorbidities, maximizing the health and quality of life of children with CP and informing guidance on coordinated care models, individual and family community involvement, and the direct and indirect costs associated with CP (5, 6).

In 2012, Novak’s team (7) conducted a systematic review of the comorbidities in CP, which revealed that 75% were in pain; 50% had an intellectual disability; 25% could not talk; 25% had epilepsy; 25% had a behavior disorder; 20% had a sleep disorder; 20% dribbled; 10% were blind; 7% were tube-fed; and 4% were deaf. However, the search language was limited to English, so there was no systematic evaluation of CP comorbidity in China. Moreover, there has yet to be a systematic review of the prevalence of CP comorbidities in China. Therefore, the purpose of this study is to conduct a systematic evaluation of the comorbidity of CP in China to provide a thorough understanding of the current state of the comorbidity of CP in China and to help medical researchers gain a comprehensive understanding of the comorbidity of CP in China.

## 2. Methods

The protocol was registered with the International Prospective Register of Systematic Reviews (PROSPERO; registration number: CRD42023406293).

### 2.1. Search strategy

Three English databases (PubMed, Embase, and Web of Science) and four Chinese databases (CNKI, Wanfang Database, VIP Database, and Chinese Biomedical Database) were systematically searched to determine all potentially relevant studies on the prevalence of comorbidities of CP reported from the establishment of the database to December 2022. Manual retrieves were conducted of the reference lists of articles considered at the full-text stage and systematic review articles on CP and comorbidities for any additional references pertinent to our systematic review. Using Endnote 20, the retrieved articles were organized, and duplicates were eliminated. The specific search strategy is shown in See Supplementary material S1.

### 2.2. Inclusion and exclusion criteria

Inclusion criteria: (i) it was directly or indirectly reported in the articles on the prevalence of each comorbidity and total comorbidity in Chinese individuals with CP. The various comorbidities included epilepsy, intellectual disability, speech disorders, hearing disorders, and vision disorders; (ii) English and Chinese articles; (iii) cross-sectional studies with raw data; (iv) Chinese and English articles with clear diagnostic criteria for CP, including the 1988 National Symposium on Pediatric CP in China (8), the 2004 National Symposium on Pediatric CP (9), the 9th National Pediatric CP Rehabilitation Academic Conference in 2006 (10), the 2015 Rehabilitation Guidelines for CP in China (11), and international diagnostic criteria for CP.

Exclusion criteria: (i) review, systematic reviews, meta-analyzes, conference abstracts, comments, or letters; (ii) studies with fewer than 50 participants were excluded to ensure that estimates reflected the intended population; (iii) the full text was not available; (iv) studies’ data were missing, duplicated, or unavailable; (v) to avoid bias in total prevalence, articles from studies that only examined populations with specific CP types was excluded; and (vi) we kept only one article for articles with overlapping collection times and data from the same region.

### 2.3. Articles screening and data extraction

Two reviewers independently reviewed the titles and abstracts of the retrieved articles to eliminate duplicate and irrelevant studies, and then independently read the full texts of the remaining articles. They then discussed their disagreements with a third senior reviewer to agree. From each article, the first author, year of publication, time of sample collection, the total number of CP individuals, number of comorbid occurrences, prevalence, age, male-to-female ratio, diagnostic criteria for CP, and diagnostic criteria for comorbidities were then separately retrieved. If more information or clarity was required, the study’s authors were contacted. Disagreements were then discussed and resolved by contacting a third senior reviewer.

### 2.4. Article quality evaluation

We used a cross-sectional/prevalence study quality rating scale recommended by the Agency for Healthcare Research and Quality (AHRQ), with 11 entries answered with “yes,” “no,” or “unclear,” and the higher the rating, the higher the quality of the articles (12), which two reviewers evaluated after discussion.

### 2.5. Data synthesis and analysis

Each comorbidity’s prevalence in CP individuals was statistically estimated using Stata 17.0 (Stata Corp, College Station, TX, United States), providing a 95% CI. To quantify the heterogeneity among the research outcomes, the Q-test and I^2^ were used. A meta-analysis was conducted after eliminating the impact of significant clinical heterogeneity and further investigating the source of heterogeneity if the level between study outcomes was high (I^2^ ≥ 50%). Conversely, a fixed-effects model was used. Sensitivity analyzes were performed using a study-by-study removal method to detect each study’s effect on the combined effect. Subgroup analysis and meta-regression analysis were used to investigate the sources of heterogeneity and differences in comorbidity prevalence between groups based on sample source of articles, gross motor function classification system (GMFCS), Chinese diagnostic criteria for CP, regional distribution, year of publication, AHRQ quality score, gender, risk factors, topographical type of CP, and clinical type of CP. Subgroup and meta-regression analysis were used to explore further the source of heterogeneity and the differences in comorbidity among different groups. The test level for the meta-analysis was set at *α* = 0.05. Moreover, a regression-based Egger’s test was used to assess the effect of small studies and the risk of publication bias. *p* < 0.05 was considered a significant bias.

## 3. Results

### 3.1. Articles screening process and results

**Figure 1:**
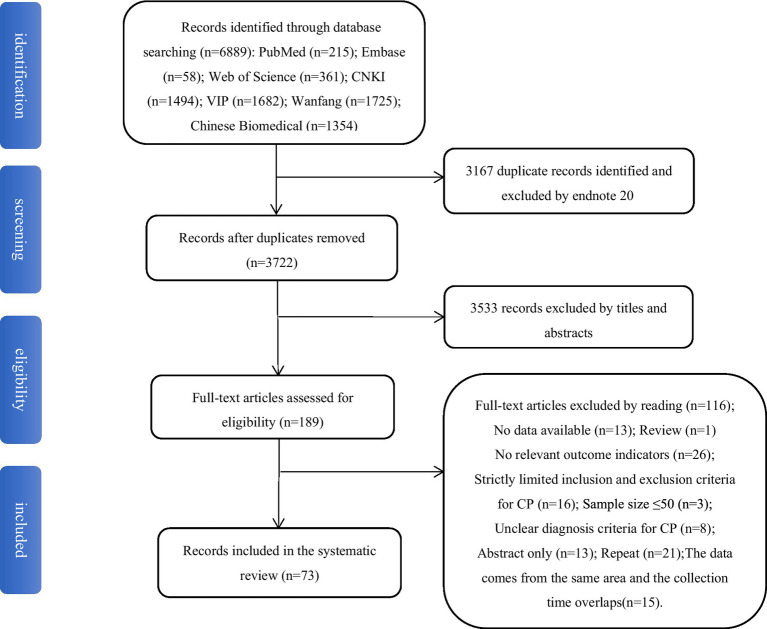
Article screening process and result.

In total, 6,889 relevant articles were searched. After 3,167 duplicates were excluded with Endnote 20, 3,533 irrelevant articles were first excluded by reading the titles and abstracts. Then 116 articles were excluded by reading the entire text and applying the inclusion and exclusion criteria. Finally, 73 articles were found to be eligible. Figure 1 depicts the article screening process.

### 3.2. Characteristics of included articles

The essential characteristics of the included articles are shown in Table 1, in which 16 articles reported total comorbidity, the most involved being epilepsy, intellectual disability, speech disorders, hearing disorders, and vision disorders, Furthermore, 56 articles reported the prevalence of epilepsy, but only 7 (14, 25, 50, 64, 75, 80, 82) mentioned the diagnostic criteria, all referring to the diagnostic criteria established by the International League Against Epilepsy (ILAF) in various years. The prevalence of intellectual disability was reported in 48 articles, with 17 articles mentioning the diagnostic methods, the most frequently used being the Gesell Developmental Scale for children ≤3 years old and the Wechsler Intelligence Scale for Children (WISC) >3 years old (16, 24, 28, 29, 33, 68, 72). Speech disorders were reported in 32 articles, with 8 articles (16, 24, 29, 33, 47, 60, 68, 80) mentioning the diagnostic methods, all of which used the Speech Symbolic Form-Indicative Content (S-S) language delay screening method and the dysarthria screening method adapted by the Chinese Rehabilitation Research Center. A total of 41 articles reported on hearing disorders, of which 19 (16, 24, 28–30, 33, 34, 36, 37, 47, 48, 55, 61, 68, 70, 73, 78, 80, 85) mentioned the diagnostic methods, all of these used brainstem auditory evoked potential (BAEP) tests. Finally, 35 articles reported on vision disorders, of which 11 (24, 28, 29, 33, 41, 47, 61, 68, 69, 71, 84) mentioned the diagnostic methods, including routine ophthalmic examinations and Visual Evoked Potential (VEP) tests. See Supplementary material S2 for details.

**Table 1:** Summary of articles.

First author	Years	Region	Sample source	Diagnostic criteria of CP	Collection time	Age range(year)	Gender(male/female)	Types of comorbidities	Prevalence of total comorbidity	AHQH score
Yu (13)	1997	Qingdao, Shandong	Medical institutions	a	1994.10 ~ 1996.05	0.25 ~ 14	59/39	(i)(ii)(iii)	-	4
Kwong (14)	1998	Hong Kong	Medical institutions	e	1990.01 ~ 1995.07	-	48/37	(i)	-	7
Liu (15)	2000	Hefei, Anhui	Medical institutions	a	1994.01 ~ 1999.01	-	50/33	(i)(ii)(iv)(v)	-	6
Cao (16)	2001	Taiyuan, Shanxi	Medical institutions	a	1992 ~ 1999	0.5 ~ 14	276/109	(i)(ii)(iii)(iv)	-	3
Dong (17)	2002	Chengdou, Sichuan	Medical institutions	a	1997.01 ~ 2001.01	0.5 ~ 11	148/114	(i)(ii)(iii)	191/262(72.9%)	6
He (18)	2002	Guangzhou, Guangdong	Medical institutions	a	2001.06 ~ 2002.03	0.5 ~ 14	87/59	(i)(ii)(iii)(iv)	64/146(43.8%)	4
Huang (19)	2002	Haikou, Hainan	Medical institutions	a	-	0.5 ~ 9	69/43	(v)	-	5
Gao (20)	2003	Henan	Population	a	-	0 ~ 6	374/208	(i)(ii)(iii)(iv)(v)	394/582(67.7%)	6
Hong (21)	2003	Jiangsu, Heilongjiang, Gansu, Sichuan, Guangxi	Population	a	-	1 ~ 6	1266/734	(i)(ii)(iii)(iv)(v)	1565/2009(77.9%)	7
Wang (22)	2004	Shanghai	Medical institutions	a	2003.01 ~ 2003.12	-	195/70	(i)(ii)(iv)(v)	-	4
Zheng (23)	2004	Taizhou, Zhejiang	Population	a	1990.01 ~ 2002.10	0 ~ 13	86/26	(i)(ii)(iii)(iv)(v)	-	3
Yao (24)	2005	Wuhan, Hubei	Medical institutions	b	2001.05 ~ 2005.05	0.5 ~ 12	67/41	(i)(ii)(iii)(iv)(v)	81/108(75.0%)	5
Lai (25)	2005	Haikou,Hainan	Medical institutions	a	1998 ~ 2003	0.3 ~ 8	64/29	(i)	-	6
Liao (26)	2005	Hechi, Guangxi	Medical institutions	a	1987.10 ~ 2003.10	0.5 ~ 14	38/17	(i)(ii)(iii)(iv)	-	4
Chan (27)	2005	Hong Kong	Population	h	2001.06 ~ 2002.06	0 ~ 22	101/80	(i)(ii)(iv)(v)	-	3
Zheng (28)	2006	Beijing	Medical institutions	a	2001.11 ~ 2005.12	0.58 ~ 11	113/82	(i)(ii)(iv)(v)	168/195(86.2%)	4
Li (29)	2006	Jinan, Shandong	Medical institutions	a	2002.10 ~ 2005.04	0 ~ 6	87/39	(i)(ii)(iii)(iv)(v)	-	4
Liu (30)	2006	Zhuhai, Guangdong	Medical institutions	a	2002.04 ~ 2005.04	0.25 ~ 4	78/40	(ii)(iii)(iv)(v)	-	3
Wang (31)	2006	Shijiazhuang, Hebei	Medical institutions	a	2002.03 ~ 2004.06	2 ~ 14	121/99	(i)(ii)	-	4
Cao (32)	2007	Tangshan, Hebei	Medical institutions	a	1990 ~ 2005.05	1 ~ 12	331/169	(i)	-	3
Zhang (33)	2007	Shenzhen, Guangdong	Medical institutions	b	2003.05 ~ 2006.05	0.67 ~ 11	37/43	(i)(ii)(iii)(iv)(v)	60/80(75%)	3
Liu (34)	2007	Wuxi, Jiangsu	Medical institutions	a	2005.7 ~ 2006.7	0.41 ~ 18	44/30	(i)(ii)(iv)(v)	-	3
Zhou (35)	2007	Lanzhou, Gansu	Medical institutions	a	2004.02 ~ 2006.12	0.33 ~ 14	186/99	(i)(ii)(iii)(v)	-	4
Wang (36)	2007	Zhengzhou, Henan	Medical institutions	a	2005.01 ~ 12	0 ~ 9	368/116	(i)(ii)(iii)(iv)	-	5
Huang (37)	2008	Haikou, Hainan	Medical institutions	b	2002.01 ~ 2007.06	0.33 ~ 9	152/106	(i)(ii)(iii)(iv)(v)	-	3
Li (38)	2008	Xuzhou, Jiangsu	Medical institutions	b	2002 ~ 2006	0 ~ 7	-	(i)	-	6
Sun (39)	2008	Rizhao, Shandong	Medical institutions	b	2004.01 ~ 2007.01	0 ~ 12	47/34	(i)(ii)(iii)(iv)(v)	69/81(85.2)	5
Hou (40)	2008	Chengde, Hebei	Medical institutions	b	1984 ~ 2000	-	148/110	(i)(ii)	-	3
Liu (41)	2008	foshan, Guangdong	Medical institutions	b	2001.03 ~ 2005.12	0.25 ~ 8	243/104	(v)	-	5
Zhang (42)	2009	Jiaozuo, Henan	Medical institutions	b	1996.06 ~ 2008.06	0.5 ~ 9	41/25	(i)	-	4
Zhou (43)	2009	Zhengzhou, Henan	Medical institutions	b	2006.01 ~ 2007.12	0 ~ 8	468/212	(i)(ii)(iii)(v)	-	4
Li (44)	2009	Shangqiu, Henan	Medical institutions	a	2006.03 ~ 2008.06	2 ~ 14	122/100	(i)(ii)	-	3
Liu (45)	2009	Taiwan	Special school	h	-	5 ~ 14	53/37	(i)(iii)	-	3
Wen (46)	2010	Zhuhai, Guangdong	Medical institutions	b	2002.01 ~ 2008.12	0 ~ 18	164/68	(i)	-	5
Wang (47)	2010	Weifang, Shandong	Medical institutions	c	2004.01 ~ 2009.12	≥1	166/124	(i)(ii)(iii)(iv)(v)	211/290(72.8%)	5
Rui (48)	2010	Hefei, Anhui	Medical institutions	b	2006 ~ 2008	0.16 ~ 13.92	288/106	(i)(ii)(iii)(iv)(v)	-	4
Chu (49)	2010	Hong Kong	Special school	f	-	≥12	36/29	(ii)	-	5
Zhu (50)	2010	Zhengzhou, Henan	Medical institutions	c	2004.03 ~ 2009.12	0.67 ~ 9	663/535	(i)	-	7
Hou (51)	2010	Qingdao, Shandong	Medical institutions	c	2007.01 ~ 2009.06	-	-	(iii)(iv)(v)	-	5
Huang (52)	2010	Taiwan	Population	h	2003.12 ~ 2004.03	≤18	206/139	(ii)	-	7
Li (53)	2011	Changchun, Jilin	Medical institutions	c	2009.03 ~ 2011.08	0.33 ~ 7	127/81	(i)(ii)(iii)(iv)(v)	-	4
Wang (54)	2011	Changsha, Hunan	Medical institutions	a	2005.01 ~ 2009.12	0.5 ~ 9	868/336	(i)(ii)(iii)(iv)	1102/1204(91.5%)	5
Tang (55)	2011	Liuzhou, Guangxi	Medical institutions	b	2009.01 ~ 2010.12	0.58 ~ 6	77/43	(iv)	-	5
Wu (56)	2011	Wuhan, Hubei	Medical institutions	b	2006.10 ~ 2008.06	1.5 ~ 3.33	75/60	(i)	-	5
Qin (57)	2011	Beijing	Medical institutions	c	-	-	760/330	(i)(ii)(iii)	-	3
Huang (58)	2012	Dongguan, Guangdong	Medical institutions	c	2006.11 ~ 2011.12	0.5 ~ 9	172/101	(i)(ii)(iii)(iv)(v)	205/273(75.1%)	3
Song (59)	2012	Zhengzhou, Henan	Medical institutions	c	2010.03 ~ 2011.07	0.33 ~ 7	224/118	(v)	-	5
Zhou (60)	2012	Zhengzhou, Henan	Medical institutions	b	2011.01 ~ 2011.12	0.25 ~ 8	334/196	(i)(ii)(iii)(iv)	-	3
Xiong (61)	2012	Changsha, Hunan	Medical institutions	c	2009.01 ~ 2012.01	0.33 ~ 8.17	86/39	(v)	-	5
Wu (62)	2012	Zibo, Shandong	Medical institutions	b	2008.01 ~ 2009.12	-	117/79	(i)(ii)(iv)(v)	155/196(79.1%)	3
Peng (63)	2013	Xiamen, Fujian	Medical institutions	c	2007.01 ~ 2012.02	0.5 ~ 6	221/209	(i)	-	7
Sun (64)	2013	Qingdao, Shandong	Medical institutions	c	2007.01 ~ 2011.06	4 ~ 6	165/50	(i)(ii)	-	5
Jia (65)	2014	Zhengzhou, Henan	Medical institutions	c	2012.01 ~ 2013.05	0.5 ~ 6	115/67	(i)(ii)(iii)(iv)(v)	161/182(88.5%)	5
Guo (66)	2014	Guangzhou, Guangdong	Medical institutions	c	2008.12 ~ 2013.12	0 ~ 12	-	(i)(ii)(iv)(v)	442/444(99.5%)	5
Li (67)	2015	Guiyang, Guizhou	Medical institutions	c	2011.05 ~ 2014.05	1 ~ 6	143/95	(i)(ii)(iv)(v)	197/238(82.8%)	5
Wang (68)	2016	Shenyang, Liaoning	Medical institutions	c	2012.01 ~ 2015.12	1.5 ~ 10	205/122	(i)(ii)(iii)(iv)(v)	305/327(93.3%)	7
Lin (69)	2016	Fuzhou, Fujian	Medical institutions	c	2014.07 ~ 12	0.67 ~ 9	93/32	(iv)	-	6
Chen (70)	2016	Nanyang, Henan	Medical institutions	b	2012.06 ~ 2013.01	1 ~ 2.25	308/216	(iv)	-	6
Li (71)	2016	Changsha, Hunan	Medical institutions	c	2015.01 ~ 06	-	170/95	(v)	-	4
Guan (72)	2017	Liaoning	Population	d	2013.01 ~ 2016.10	3 ~ 14	1058/265	(i)(ii)(iii)(v)	-	5
Shu (73)	2017	Shiyan, Hubei	Medical institutions	b	-	-	65/35	(iv)	-	3
Xie (74)	2017	Huangshi, Hubei	Medical institutions	c	2013.05 ~ 2016.05	4 ~ 6	/	(i)(ii)	-	7
Zhang (75)	2017	Zhengzhou, Henan	Medical institutions	d	2013.12 ~ 2015.05	<5	125/48	(i)(ii)	-	7
He (76)	2017	Nationwide	Population	h	2006.04 ~ 05	<17	266,591/140474	(ii)(iv)(v)	-	7
Ke (77)	2018	Hangzhou, Zhejiang,	Medical institutions	d	2011.01 ~ 2016.12	0 ~ 6	183/117	(i)(ii)(iii)(iv)	-	4
Yang (78)	2018	Taian, Shandong	Medical institutions	d	2015.05 ~ 2017.05	0.5 ~ 4	61/51	(iv)	-	6
Chiang (79)	2019	Taiwan	Population	f	2010 ~ 2011	0.08 ~ 19	-	(i)(ii)(iii)(iv)	-	6
Yuan (80)	2020	Henan	Population	d	2014.05 ~ 2016.07	0.41 ~ 20.3	936/421	(i)(ii)(iii)(iv)(v)	-	7
Wang (81)	2022	Nanyang, Henan	Medical institutions	j	2019.01 ~ 2020.06	0.25 ~ 18	64/43	(i)	-	7
Niu (82)	2022	Zhengzhou, Henan	Medical institutions	d	2019.01 ~ 2022.02	0.25 ~ 18	421/209	(i)	-	7
Yang (83)	2022	Yili, Xinjiang	Medical institutions	d	2018.06 ~ 2020.06	<14	232/173	(i)(ii)	-	4
Lin (84)	2022	Kashi, Xinjiang	Population	d	2018.1	0.8 ~ 12	104/72	(v)	-	6
Zhu (85)	2022	Jiamusi, Heilongjiang	Medical institutions	d	2018.12 ~ 2019.11	-	-	(iv)	-	5

Diagnostic criteria of CP: a. National Symposium on Cerebral Palsy in Children in 1988; b. National Symposium on Pediatric Cerebral Palsy in 2004; c. The 9th National Conference on Rehabilitation of Children with Cerebral Palsy in 2006; d. Chinese Rehabilitation Guideline for Cerebral Palsy in 2015; e. Terminology and classification of cerebral palsy in 1964 (86); f. Proposed definition and classification of cerebral palsy in 2005 (87); g. WHO Classification of Functioning, Disability and Health; h. Cerebral palsy in 2004 (88); j. Expert consensus on etiological diagnosis strategy of cerebral palsy in 2019. Comorbidities: (i) Epilepsy; (ii) Intellectual Disability; (iii) Speech Disorders; (iv) Hearing Disorders; (v) Vision Disorders.

### 3.3. Article quality evaluation

After the quality evaluation, each article was found to have a score between 3 and 7, with 16 articles having scores of 3, 15 articles scores of 4, 20 articles scores of 5, 10 articles scores of 6, and 12 articles scores of 7. The mean score was 4.82, showing that the results had a high risk of bias, and the specific article quality evaluation results are shown in Supplementary material S3.

### 3.4. Results of meta-analysis and publication bias

**Figure 2:**
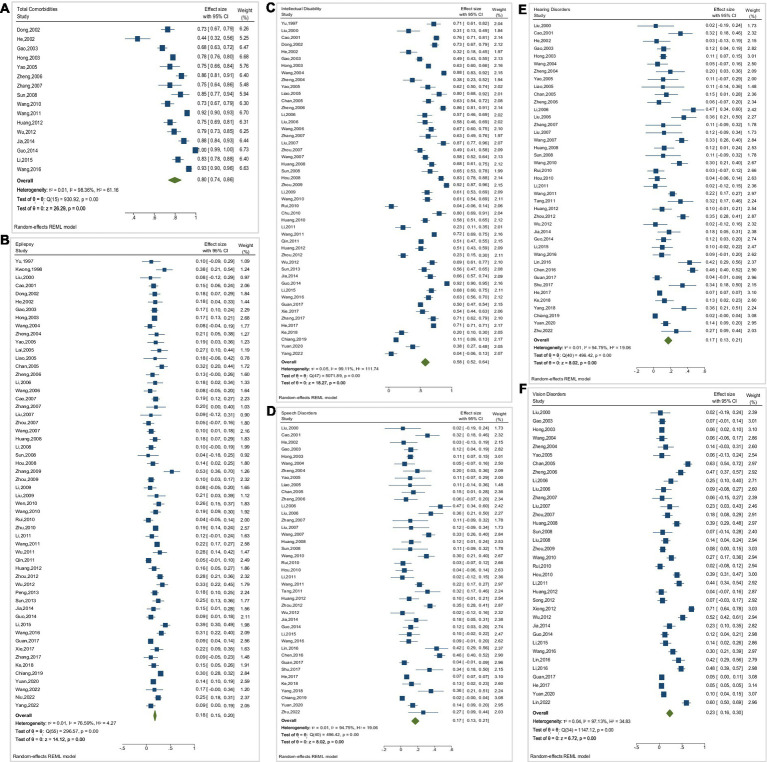
Forest plots of meta-analysis of the prevalence of comorbidities. **(A)** Total Comorbidities, **(B)** Epilepsy, **(C)** Intellectual Disability, **(D)** Speech Disorders, **(E)** Hearing Disorders, **(F)** Vision Disorders.

The meta-analysis results showed high inter-study heterogeneity (I^2^ ≥ 50%) for all outcome indicators, and therefore all were analyzed using a random-effects model. Articles on the prevalence of total comorbidity involved a total of 6,617 individuals with CP and showed *I*^2^ = 98.36, with a prevalence of 79.7% (95% CI: 73.8–85.7%) and some publication bias (*p* < 0.001). Articles on the prevalence of epilepsy involved a total of 29,020 individuals with CP and showed *I*^2^ = 76.59, with a prevalence of 17.9% (95% CI: 15.4–20.4%) and some publication bias (*p* = 0.002). The articles on the prevalence of intellectual disability involved 430,933 individuals with CP and showed *I*^2^ = 99.11, with a prevalence of 58.0% (95% CI: 51.8–64.3%) and some publication bias (*p* = 0.026). The articles on the prevalence of speech disorders involved 21,426 individuals with CP and showed an *I*^2^ = 97.14, with a prevalence of 48.0% (95% CI: 41.6–54.4%) and no significant publication bias (*p* = 0.135). The articles on the prevalence of hearing disorders involved 429,032 individuals with CP and showed an *I*^2^ = 94.75, with a prevalence of 17.2% (95% CI: 13.0–21.4%)and some publication bias (*p* < 0.001). The articles on the prevalence of vision disorders involved 419,150 individuals with CP and showed *I*^2^ = 97.13, with a prevalence of 23.1% (95% CI: 16.3–29.8%) and some publication bias (*p* < 0.001). These results are shown in Table 2 and Figure 2.

**Table 2:** Summarizes the meta-analysis results and Egger’s test of comorbidities.

Comorbidity	Number of articles	Number of CP	Event	Heterogeneity		(95% CI)		Prevalence(%)	Publication bias (Egger’s test)	
				*I*^2^(%)	*p* value					
									*t*	*P* value
Total Comorbidities	16	6,617	5,370	98.36	<0.001	73.8	85.7	79.7	−5.15	<0.001
Epilepsy	56	29,020	5,833	76.59	<0.001	15.4	20.4	17.9	−3.28	0.002
Intellectual disability	48	430,933	298,976	99.11	<0.001	51.8	64.3	58.0	−2.30	0.026
Speech disorders	32	21,426	7,687	97.14	<0.001	41.6	54.4	48.0	1.54	0.135
Hearing disorders	41	429,032	30,740	94.75	<0.001	13.0	21.4	17.2	3.90	<0.001
Vision disorders	35	419,150	22,371	97.13	<0.001	16.3	29.8	23.1	4.66	<0.001

### 3.5. Sensitivity analysis

A stepwise elimination method was used to investigate the origin of heterogeneity and the results were comparable to the overall detection rate. The following outcome indicators had better stability: total comorbidity (78.4–81.9%), epilepsy (17.4–18.2%), intellectual disability (57.1–59.0%), speech disorders (47.1–49.4%), hearing disorders (16.5–17.9%), and vision disorders (21.6–23.7%). See Supplementary material S4 for details.

### 3.6. Subgroup analysis and meta-regression analysis

**Figure 3:**
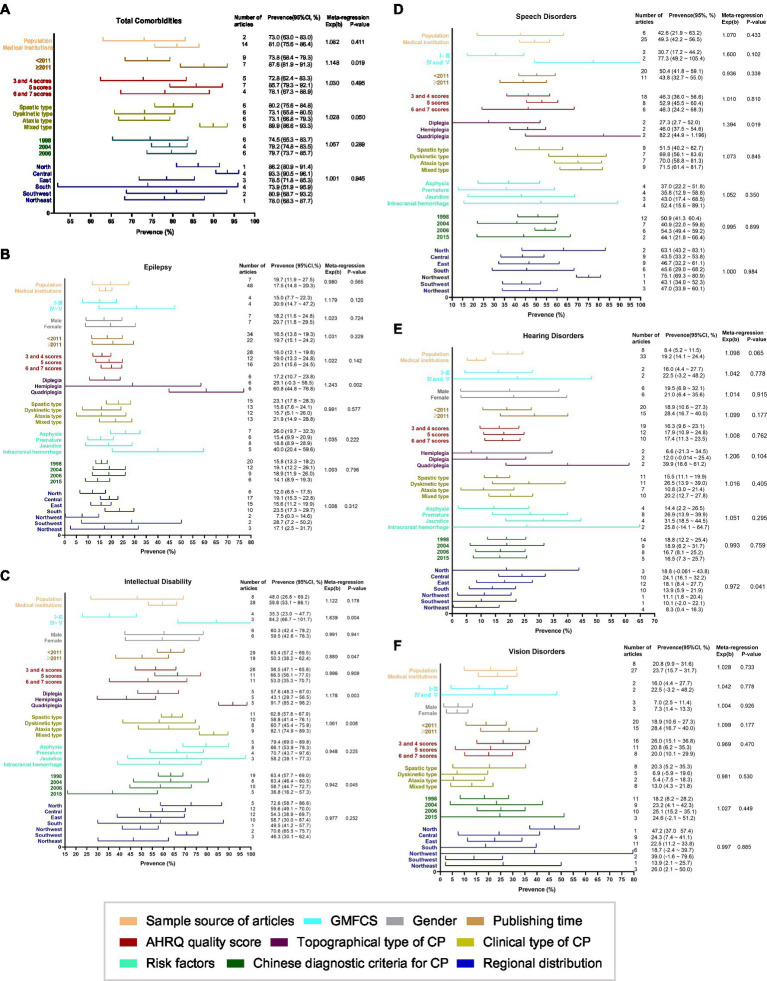
Subgroup analysis and meta-regression analysis of the prevalence of comorbidities. **(A)** Total Comorbidities, **(B)** Epilepsy, **(C)** Intellectual Disability, **(D)** Speech Disorders, **(E)** Hearing Disorders, **(F)** Vision Disorders.

By subgroup analysis and meta-regression analysis, it was found that for total comorbidities, the publishing time was the source of heterogeneity (*p* = 0.019). For epilepsy prevalence, the topographical type of CP was the source of heterogeneity (*p* = 0.002). Regarding intellectual disability, the primary sources of heterogeneity were GMFCS (*p* = 0.004), publishing time (*p* = 0.047), diagnostic criteria for CP (*p* = 0.045), clinical type (*p* = 0.004), and topographical type (*p* = 0.008). For speech disorders, the topographical type of CP was the primary source of heterogeneity (*p* = 0.019). For hearing disorders, the region was the primary source of heterogeneity (*p* = 0.041). These results are shown in Figure 3.

## 4. Discussion

This study collated and summarized articles on the comorbidity of CP in China over the past 25 years. The results showed that, after excluding articles that did not meet the criteria, 73 articles studied the comorbidity of CP. The sample size of the included articles was extensive. It covered most regions of China, which could reflect the characteristics, development trends, and distribution of comorbidity in CP of China to some extent. However, some of the articles were missing relevant information, and the unequal number of articles included in the subgroup analysis may have affected the comprehensiveness of the results.

Our analysis of CP comorbidity showed that the prevalence of intellectual disability was 58.0%, speech disorders were 48.0%, epilepsy was 17.9%, hearing disorders were 17.2%, and vision disorders were 23.1%. This was different from a population-based study in Australia, which showed that 48% had an intellectual disability, 61% had speech disorders, 28% had epilepsy before the age of 5, 12% had hearing disorders, and 36% had vision disorders (89). We presumed that this was due to differences in ethnicity, geographic and natural environment, and level of health care. In addition, the results showed that the prevalence of epilepsy in this study was lower than the prevalence of 30.5% in Europe and 41% in the United States (90, 91). The reason may be the negligence of early diagnosis in China in the early years, which leads to the failure to diagnose epilepsy in some children with CP early in life because epilepsy occurs mainly in children with CP within 1 year of age and is a marker for early determination of the severity of CP (92, 93). Early diagnosis is vital for rapid etiological assessment, providing early intervention, and preventing complications. The more severe the brain insult, the higher the likelihood of comorbidities (94). In addition, compared to other comorbidities, epilepsy is not directly observed by assessment. It requires a seizure for diagnosis, and most of the included articles obtained the prevalence of epilepsy by reviewing medical history data. Because of the lack of awareness of epilepsy among most Chinese parents, combined with some motor deficits in CP, such as dyskinetic CP with complex partial seizure, parents may consider this a manifestation of CP dyskinesia. When taking a medical history, parents will not think that their child has epilepsy, resulting in an underdiagnosis (93) which may make the prevalence of epilepsy somewhat underestimated. Furthermore, most CP individuals in China are only admitted for short hospital stays and receive rehabilitation in outpatient clinics, making it difficult to accurately and timely document epilepsy when it occurs.

In this study, the prevalence of total comorbidity, epilepsy, hearing disorders, and vision disorders increased after 2011 compared to before 2011. We assume this may be because the diagnostic criteria, tools, and equipment used to detect these comorbidities in China improved and became more sensitive. The idea of early diagnosis is gradually gaining importance. More comorbidities of CP are being correctly diagnosed. However, diagnostic scales for intellectual disability (Gesell Developmental Scale for ≤3 years old, WISC for >3 years old) and speech disorders (S-S speech delay test and dysarthria test adopted by China Rehabilitation Research Center) are the diagnostic tools that most medical institutions in China have used since their introduction from abroad, and the diagnostic criteria have not changed significantly. Moreover, increasing attention and the implementation of early intervention in China may be the reason for the decrease in the prevalence of epilepsy, intellectual disability, and speech disorders in CP after 2011.

Among our included articles, the AHRQ scores ranged from 3 to 7, with extensive range and variable quality. We performed subgroup analyzes based on the AHRQ scores to explore the prevalence of CP comorbidities in different quality articles. This study found that compared with low- and middle-quality articles, the prevalence of most comorbidities in high-quality articles with scores of 6 and 7 was closer to the totalized prevalence in Table 2, suggesting that the totalized results were stable. In contrast, the prevalence of most comorbidities was higher in the articles that scored 5 than in the other two groups, which was caused by the higher prevalence of comorbidities in the multiple articles included.

In this study, we divided it into the clinical type and topographical type for subgroup analysis according to the CP typing criteria of most of the included articles. The results showed that the prevalence of mixed type in clinical type and quadriplegia in topographical type was high. These two types are characterized by relatively severe brain insult, and their GMFCS is often class IV and V. The subgroup analysis of GMFCS found that the prevalence of comorbidities in GMFCS classes IV and V was higher than that in class I-III, which verified that individuals with severe CP were more likely to have comorbidities. Delacy et al. (89) showed that as the GMFCS level increased, the proportion of children with two or more severely related impairments increased from 3% in GMFCS class I to 73% in GMFCS class V. The prevalence of comorbidities increased, with quadriplegia being the group with the highest comorbidities in CP. In addition, we found a higher prevalence of comorbidities in hospital-based studies than in population-based studies, possibly because hospital-based studies tended to include more severe CP individuals. In conclusion, we speculate that the prevalence of comorbidities is related to the severity of CP. Also, it was discovered that the position of the brain insult impacted the occurrence of comorbidity in CP. For example, we found that in terms of the prevalence of epilepsy, intellectual disability, and vision disorders, the spastic type with predominantly cortical brain insult was the highest. In contrast, the dyskinetic type with extrapyramidal insult and the ataxia type with predominantly cerebellar insult were low. However, the prevalence of speech disorders was high in the dyskinetic and ataxia types because the dyskinetic type has dystonia due to extrapyramidal insult, which leads to this dysarthria. The cerebellum mainly controls language processing tasks, so the insult leads to a series of speech disorders.

There is some difference in the prevalence of CP comorbidity by risk factors; for instance, the results of the subgroup analysis show that intracranial hemorrhage was a high prevalence factor for epilepsy. This is because brain insult is more severe in CP with intracranial hemorrhage, which can cause the release of numerous inflammatory factors in the child’s brain cavity, damaging the blood–brain barrier, which can cause functional damage to the child’s ventricles as well as peripheral tissues. Furthermore, it can cause a functional imbalance in glutamate metabolism in the brain, allowing synchronous glutamate firing in the hippocampal neural network and increasing the risk of epilepsy (81). However, risk factors for CP are often multifactorial and confounded, and different risk factors can combine to cause CP, so the results still need to be interpreted cautiously.

To present, the diagnostic criteria for CP have been revised five times in China, with some differences in the diagnostic criteria for CP after each revision, so we analyzed the past four CP guidelines by subgroups (excluding the latest revised Chinese CP guidelines in 2022 (1), which are not currently widely used). The difference in the prevalence of comorbidity was found to be greater in the group based on the 2015 guidelines than in the other groups. This result may be due to a significant change in the diagnostic criteria of the 2015 Chinese CP Guidelines Group. The hypotonic type was removed as a subtype of CP, which was considered to be a transitional form of other CP subtypes, and the type was only defined as a developmental delay in the diagnosis. Furthermore, the low number of articles using the 2015 guideline as the diagnostic criteria may be one of the reasons.

The prevalence of CP comorbidity varies widely among geographic subdivisions. This difference is related to the fact that China is a multi-ethnic country with unbalanced levels of education, economic development, regional healthcare conditions, and CP prevention and treatment measures and capabilities, among other factors (95). In economically developed coastal regions such as South China, where regional development is rapid and the coverage of rehabilitation services is more extensive, early diagnosis and intervention are emphasized and implemented. Hence, the diagnosis rate of epilepsy was higher (28.7%). The prevalence of comorbidities such as hearing disorders (13.9%), vision disorders (18.7%), and speech disorders (45.6%) was lower than in other regions. Although the central and western regions are vast, they are relatively slow to develop and are limited by lower economic conditions and healthcare levels. Prevention and management of CP comorbidities are lacking (96), and the prevalence of comorbidities was higher. However, more studies are needed to verify this in the future because of the small number of included articles in this group.

In addition, the results of this study are more reflective of the prevalence rate of comorbidity of CP in medical institutions, as 87.2% of the included studies were medical institution-based studies. Theoretically, patients with CP in medical institution-based studies are more likely to be admitted to rehabilitation for severe conditions. In contrast, patients with CP who have relatively mild symptoms and are from low-income families may not be admitted to rehabilitation, thus, the final results may overestimate the comorbidity of CP (97). In 1998, Surveillance of Cerebral Palsy in Europe (SCPE) became the world’s first CP surveillance network, consisting of 14 CP management centers in eight countries. By 2018, it had expanded to 23 centers in 20 countries, registering 21,043 children with CP born from 1976 to 2018. SCPE coordinated the European CP registry management centers to cooperate and develop a central database to provide uniform standards and criteria for collaborative research (98). Although Japan and Korea have not established a CP surveillance system, nationwide health insurance systems covering the whole country can monitor the prevalence of CP, understand comorbidities and risk factors, and provide prevention strategies, thus serving as a database for policy development and playing an essential role in the prevention, treatment, and rehabilitation of CP (99, 100). In China, only some provinces and cities have built CP child detection systems and information platforms, and no reports have established national or multi-regional collaborative CP monitoring systems and information platforms (95). So far, only Henan Province has published relevant articles through data from the CP child registration management monitoring network built in 2020 (80), and no related research has been reported in other provinces and cities. In China, one can consult SCPE and the Australian Cerebral Palsy Register (ACPR) (101) as information platforms to establish a standard of unified data collection table. For example, through many parts of the country, at hospitals, rehabilitation centers, and special education schools, CP individuals are registered through an online and/or paper-based registration system, which specialized agencies and personnel then administer. However, it is still difficult to establish a CP registry in China because China has a wide geographical area, uneven economic development, and numerous institutions accepting CP children, so it needs gradual development and establishment. It is of great significance to establish a sound national CP register and understand the epidemic characteristics and trends of CP over time through a network monitoring system and information data platforms for the formulation of effective prevention and treatment strategies for CP.

The limitations of this study are as follows. (i) This study is a meta-analysis of a single rate group, with enormous heterogeneity among studies. (ii) The included CP cases were of varying severity, and the different confounding risk factors and severity of CP may produce some differences in the results. (iii) The inclusion of an extensive age range and low age may have some influence on the final results, due to the reason that some comorbidities often develop with age, and younger children with CP comorbidities may be difficult to diagnose (102), so the older the CP patient is at the time of recording, the less likely it is that the diagnosis will be missed (103). (iv) The substantial between-study heterogeneity limits the interpretability and clinical applicability of the reported prevalence figures.

In conclusion, the prevalence of CP comorbidity is high in China. Comorbidity is related to brain insult characteristics, severity, and risk factors. It has a particular relationship with regional economic development and medical and health levels. Future studies on the prevention of CP comorbidity to improve the survival and quality of life of CP individuals should pay attention to these factors.

## Data availability statement

The original contributions presented in the study are included in the article/Supplementary material, further inquiries can be directed to the corresponding authors.

## Author contributions

CG, JG, and SZ were responsible for the conception and design of the article, the revision of the article, and the overall responsibility of the article. CG, LF, and MZ were responsible for the implementation and feasibility analysis of the study and statistical processing. BL, AL, and SC were responsible for the articles search, data collection, and organization. CG, SZ, LF, and XL were responsible for analyzing and interpreting the results. CG, JG, and AL were responsible for the writing and proofreading the article. HL and XQ participated in the revision of the article.

## Supplementary material

The Supplementary material for this article can be found Available at: https://www.frontiersin.org/articles/10.3389/fneur.2023.1233700/full#supplementary-material
