# Supplementary material for: Prevalence of cerebral palsy comorbidities in China: a systematic review and meta-analysis
Source: Front Neurol. 2023 Sep 28;14:1233700. doi: 10.3389/fneur.2023.1233700 (PMC10568468; doi:10.3389/fneur.2023.1233700)
Supplement: Supplementary file 1 [file Data_Sheet_1.docx]

| **Table 1. The search strategy for Pubmed** | |
| --- | --- |
| **steps** | **Search Formulation** |
| #1 | (("Cerebral Palsy"[Mesh]) OR ("Cerebral Pals*"[Title/Abstract])) OR ("Little Disease"[Title/Abstract]) |
| #2 | ((Complication[Title/Abstract]) OR (Comorbidit*[Title/Abstract])) OR (Morbidit*[Title/Abstract]) |
| #3 | (((((Epilepsy[Mesh]) OR (Seizure*[Mesh])) OR (Aura*[Title/Abstract])) OR (Epileps*[Title/Abstract])) OR (Seizure*[Title/Abstract])) OR (Convulsion[Title/Abstract]) |
| #4 | ((("Intellectual Disability"[Mesh]) OR ("Mental Retardation"[Title/Abstract])) OR ("Intellectual Disability"[Title/Abstract])) OR ("Mental Subnormality"[Title/Abstract]) |
| #5 | (((("Speech Disorder*[Title/Abstract]") OR ("Language Disorders"[Title/Abstract])) OR ("Language Impairment"[Title/Abstract])) OR (Dysarthria[Title/Abstract])) OR ("Articulation Disorder*"[Title/Abstract]) |
| #6 | (((("Hearing disorder*"[Title/Abstract]) OR ("Hearing Impairment"[Title/Abstract])) OR ("Hearing Loss"[Title/Abstract])) OR (Hypoacusis[Title/Abstract])) OR (Deaf*[Title/Abstract]) |
| #7 | (((("Vision Disorder*"[Title/Abstract]) OR ("Visual Disorder*"[Title/Abstract])) OR ("Vision Impairment"[Title/Abstract])) OR ("Visual Disturbance"[Title/Abstract])) OR (Blind[Title/Abstract]) |
| #8 | #2 OR #4 OR #5 OR #6 OR #7 |
| #9 | (((China) OR (Chinese)) OR (Taiwan)) OR (Hong Kong) |
| #10 | #1 AND #8 AND #9 |

| **Table 2. The search strategy for Web of Science** | |
| --- | --- |
| **Steps** | **Search Formulation** |
| #1 | TS=("Cerebral Pals*") OR TS=("Little Disease") |
| #2 | ((TS=(Complication)) OR TS=(Comorbidit*)) OR TS=(Morbidit*) |
| #3 | (((TS=(Epileps*)) OR TS=(Seizure*)) OR TS=(Aura*)) OR TS=(Convulsion) |
| #4 | ((TS=("Intellectual Disability")) OR TS=("Mental Retardation")) OR TS=("Mental Subnormality") |
| #5 | ((((TS=("Speech Disorder*")) OR TS=("Language Disorder*")) OR TS=("Language Impairment")) OR TS=(Dysarthria)) OR TS=("Articulation Disorder*") |
| #6 | ((((TS=("Hearing Disorder*")) OR TS=("Hearing Loss")) OR TS=(Hypoacusis)) OR TS=(Deaf*)) OR TS=("Hearing Impairment") |
| #7 | ((((TS=("Vision Disorder*")) OR TS=("Visual Disorder*")) OR TS=("Vision Impairment")) OR TS=("Visual Disturbance")) OR TS=(Blind) |
| #8 | #2 OR #3 OR #4 OR #5 OR #6 OR #7 |
| #9 | (((ALL=(China)) OR ALL=(Chinese)) OR ALL=(Taiwan)) OR ALL=(Hong Kong) |
| #10 | #1 AND #8 AND #9 |

| **Table 3. The search strategy for Embase** | |
| --- | --- |
| **Steps** | **Search Formulation** |
| #1 | 'Cerebral Pals*':ab,ti OR 'Little Disease':ab,ti |
| #2 | Complication:ab,ti OR Comorbidit*:ab,ti OR Morbidit*:ab,ti |
| #3 | Epileps*:ab,ti OR Seizure*:ab,ti OR Aura*:ab,ti OR Convulsion* |
| #4 | 'Speech Disorder*':ab,ti OR 'Language Disorder*':ab,ti OR 'Language Impairment':ab,ti OR Dysarthria:ab,ti OR 'Articulation Disorder*':ab,ti |
| #5 | 'Intellectual Disability':ab,ti OR 'Mental Retardation':ab,ti OR 'Mental Subnormality':ab,ti |
| #6 | 'Hearing Disorder*':ab,ti OR 'Hearing Impairment':ab,ti OR 'Hearing Loss':ab,ti OR Hypoacusis:ab,ti OR Deaf*:ab,ti |
| #7 | 'Vision Disorder*':ab,ti OR 'Visual Disturbance':ab,ti OR 'Vision Impairment':ab,ti OR 'Visual Disorder*':ab,ti OR Blind:ab,ti |
| #8 | #2 OR #3 OR #4 OR #5 OR #6 OR #7 |
| #9 | China OR Chinese OR Taiwan OR Hong Kong OR Aomen |
| #10 | #1 AND #8 AND #9 |
