# Supplementary material for: Prevalence of cerebral palsy comorbidities in China: a systematic review and meta-analysis
Source: Front Neurol. 2023 Sep 28;14:1233700. doi: 10.3389/fneur.2023.1233700 (PMC10568468; doi:10.3389/fneur.2023.1233700)
Supplement: Supplementary file 2 [file Data_Sheet_2.docx]

**Table 1. Summary of articles reporting epilepsy-related information**

| **No.** | **author** | **years** | **n** | **event** | **Prevalence (%)** | **Diagnostic criteria for epilepsy** |
| --- | --- | --- | --- | --- | --- | --- |
| 14 | Yu | 1997 | 98 | 10 | 10.2 | - |
| 15 | Kwong | 1998 | 85 | 32 | 37.6 | International League Against Epilepsy (ILAF) in 1993 |
| 16 | Liu | 2000 | 83 | 7 | 8.4 | - |
| 17 | Cao | 2001 | 385 | 58 | 15.1 | - |
| 18 | Dong | 2002 | 262 | 46 | 17.6 | - |
| 19 | He | 2002 | 146 | 27 | 18.5 | - |
| 21 | Gao | 2003 | 582 | 99 | 17.0 | - |
| 22 | Hong | 2003 | 2009 | 341 | 17.0 | - |
| 23 | Wang | 2004 | 265 | 20 | 7.5 | - |
| 24 | Zheng | 2004 | 112 | 24 | 21.4 | - |
| 25 | Yao | 2005 | 108 | 21 | 19.4 | - |
| 26 | Lai | 2005 | 93 | 25 | 26.9 | ILAF in 1981 and 1989 |
| 27 | Liao | 2005 | 55 | 10 | 18.2 | - |
| 28 | Chan | 2005 | 181 | 58 | 32.0 | - |
| 29 | Zheng | 2006 | 195 | 25 | 12.8 | - |
| 30 | Li | 2006 | 126 | 23 | 18.3 | - |
| 32 | Wang | 2006 | 220 | 17 | 7.7 | - |
| 33 | Cao | 2007 | 500 | 97 | 19.4 | - |
| 34 | Zhang | 2007 | 80 | 16 | 20 | - |
| 35 | Liu | 2007 | 74 | 7 | 9.5 | - |
| 36 | Zhou | 2007 | 285 | 13 | 4.6 | - |
| 37 | Wang | 2007 | 484 | 48 | 9.9 | - |
| 38 | Huang | 2008 | 258 | 47 | 18.2 | - |
| 39 | Li | 2008 | 362 | 35 | 9.7 | - |
| 40 | Sun | 2008 | 81 | 3 | 3.7 | - |
| 41 | Hou | 2008 | 258 | 35 | 13.6 | - |
| 43 | Zhang | 2009 | 66 | 35 | 53.0 | - |
| 44 | Zhou | 2009 | 680 | 66 | 9.7 | - |
| 45 | Li | 2009 | 222 | 17 | 7.7 | - |
| 46 | [Liu](https://www.webofscience.com/wos/author/record/28777125) | 2009 | 90 | 19 | 21.1 | - |
| 47 | Wen | 2010 | 232 | 60 | 25.9 | - |
| 48 | Wang | 2010 | 290 | 56 | 19.3 | - |
| 49 | Rui | 2010 | 394 | 17 | 4.3 | - |
| 51 | Zhu | 2010 | 1198 | 231 | 19.3 | ILAF in 2001 |
| 54 | Li | 2011 | 208 | 24 | 11.5 | - |
| 55 | Wang | 2011 | 1204 | 268 | 22.3 | - |
| 63 | Wu | 2011 | 135 | 38 | 28.1 | - |
| 58 | [Qin](http://med.wanfangdata.com.cn/Author/General/A000035461) | 2011 | 1090 | 50 | 4.6 | - |
| 59 | Huang | 2012 | 273 | 44 | 16.1 | - |
| 61 | Zhou | 2012 | 530 | 151 | 28.5 | - |
| 63 | Wu | 2012 | 196 | 65 | 33.2 | - |
| 64 | Peng | 2013 | 522 | 92 | 17.6 | - |
| 65 | Sun | 2013 | 215 | 53 | 24.7 | ILAF in 1981 |
| 66 | Jia | 2014 | 182 | 27 | 14.8 | - |
| 67 | Guo | 2014 | 444 | 42 | 9.5 | - |
| 68 | Li | 2015 | 238 | 94 | 39.5 | - |
| 69 | Wang | 2016 | 327 | 101 | 30.9 | - |
| 73 | Guan | 2017 | 1323 | 120 | 9.1 | - |
| 75 | Xie | 2017 | 183 | 40 | 21.9 | - |
| 76 | Zhang | 2017 | 173 | 16 | 9.2 | ILAF in 2014 |
| 78 | Ke | 2018 | 300 | 46 | 15.3 | - |
| 80 | Chiang | 2019 | 8419 | 2510 | 29.8 | - |
| 81 | Yuan | 2020 | 1357 | 196 | 14.4 | ILAF in 2014 |
| 82 | Wang | 2022 | 107 | 18 | 16.8 |  |
| 83 | Niu | 2022 | 630 | 155 | 24.6 | ILAF in 2017 |
| 84 | [Yang](http://qikan.cqvip.com/Qikan/Search/Index?key=A=%e6%9d%a8%e4%b9%90) | 2022 | 405 | 38 | 9.4 | - |
| Total | - | - | 29020 | 5833 | - | - |

**Table 2. Summary of articles reporting intellectual disability related information**

| **No.** | **author** | **years** | **n** | **event** | **Prevalence (%)** | **Diagnostic criteria for intellectual disability** |
| --- | --- | --- | --- | --- | --- | --- |
| 14 | Yu | 1997 | 98 | 70 | 71.4 | - |
| 16 | Liu | 2000 | 83 | 26 | 31.3 | - |
| 17 | Cao | 2001 | 385 | 291 | 75.6 | Gesell Developmental Scale was used for ≤ 3 years old, and China-Bine Scale was used for > 3 years old |
| 18 | Dong | 2002 | 262 | 191 | 72.9 | - |
| 19 | He | 2002 | 146 | 46 | 31.5 | - |
| 21 | Gao | 2003 | 582 | 285 | 49 | - |
| 22 | Hong | 2003 | 2009 | 1265 | 63 | - |
| 23 | Wang | 2004 | 265 | 232 | 87.5 | - |
| 24 | Zheng | 2004 | 112 | 42 | 37.5 | - |
| 25 | Yao | 2005 | 108 | 67 | 62 | Wechsler Intelligence Scale (WISC) |
| 27 | Liao | 2005 | 55 | 44 | 80 | - |
| 28 | Chan | 2005 | 181 | 114 | 63 | - |
| 29 | Zheng | 2006 | 195 | 168 | 86.2 | Gesell developmental scale |
| 30 | Li | 2006 | 126 | 72 | 57.1 | Gesell developmental scale |
| 31 | Liu | 2006 | 118 | 68 | 57.6 | - |
| 32 | Wang | 2006 | 220 | 148 | 67.3 | - |
| 34 | Zhang | 2007 | 80 | 50 | 62.5 | WISC |
| 35 | Liu | 2007 | 60 | 52 | 86.7 | Neuropsychological test |
| 36 | Zhou | 2007 | 285 | 141 | 49.5 | - |
| 37 | Wang | 2007 | 484 | 281 | 58.1 | - |
| 38 | Huang | 2008 | 258 | 176 | 68.2 | Pediatric intelligence development screening test (DDST) |
| 40 | Sun | 2008 | 81 | 53 | 65.4 | - |
| 41 | Hou | 2008 | 258 | 214 | 82.9 | - |
| 44 | Zhou | 2009 | 180 | 165 | 91.7 | Pediatric intelligence development screening test (DDST) |
| 45 | Li | 2009 | 222 | 135 | 60.8 | - |
| 48 | Wang | 2010 | 290 | 178 | 61.4 | Diagnostic scale of neuropsychological development in children aged 0-6 years |
| 49 | Rui | 2010 | 394 | 16 | 4.1 | - |
| 50 | Chu | 2010 | 65 | 52 | 80 | - |
| 53 | Huang | 2010 | 345 | 200 | 58 | - |
| 54 | Li | 2011 | 208 | 47 | 22.6 | - |
| 55 | Wang | 2011 | 1204 | 868 | 72.1 | - |
| 58 | [Qin](http://med.wanfangdata.com.cn/Author/General/A000035461) | 2011 | 1090 | 559 | 51.3 | - |
| 59 | Huang | 2012 | 273 | 139 | 50.9 | - |
| 61 | Zhou | 2012 | 530 | 121 | 22.8 | - |
| 63 | Wu | 2012 | 196 | 135 | 68.9 | - |
| 65 | Sun | 2013 | 210 | 118 | 56.2 | WISC-RC |
| 66 | Jia | 2014 | 182 | 120 | 65.9 | - |
| 67 | Guo | 2014 | 444 | 410 | 92.3 | - |
| 68 | Li | 2015 | 238 | 161 | 67.6 | - |
| 69 | Wang | 2016 | 327 | 206 | 63 | Gesell Developmental Scale for ≤ 3 years old and WISC for > 3 years old were used. |
| 73 | Guan | 2017 | 1323 | 666 | 50.3 | Gesell developmental scale |
| 75 | Xie | 2017 | 183 | 98 | 53.6 | WISC-RC |
| 76 | Zhang | 2017 | 173 | 122 | 70.5 | Chinese Classification and Diagnostic Criteria of Mental Disorders-3 (CCMD-3) |
| 77 | He | 2017 | 407065 | 289242 | 71.1 | DDST and WISC of children aged 0-6 years, 7-16 years and over 17 years old |
| 78 | Ke | 2018 | 300 | 61 | 20.3 | - |
| 80 | Chiang | 2019 | 8419 | 964 | 11.5 | - |
| 81 | Yuan | 2020 | 216 | 82 | 38 | The fifth edition of the Diagnostic and Statistical Manual of Mental Disorders (DSM-V) diagnostic criteria was used for children over 5 years old. The inclusion criteria for 4 to 5 years old were Wechsler Intelligence Test IQ < 70, and the standard score of infant-junior high school students ' social adaptability ≤ 8. |
| 84 | [Yang](http://qikan.cqvip.com/Qikan/Search/Index?key=A=%e6%9d%a8%e4%b9%90) | 2022 | 405 | 15 | 3.7 | Pediatric neuropsychological development checklist |
| Total | - | - | 430933 | 298976 | - | - |

**Table 3. Summary of articles reporting speech disorder related information**

| **No.** | **author** | **years** | **n** | **event** | **Prevalence (%)** | **Diagnostic criteria for speech disorders** |
| --- | --- | --- | --- | --- | --- | --- |
| 14 | Yu | 1997 | 98 | 76 | 77.6 | - |
| 17 | Cao | 2001 | 189 | 139 | 73.5 | S-S speech disorders test and dysarthria test adapted by China Rehabilitation Research Center |
| 18 | Dong | 2002 | 262 | 113 | 43.1 | - |
| 19 | He | 2002 | 146 | 43 | 29.5 | - |
| 21 | Gao | 2003 | 582 | 206 | 35.4 | - |
| 22 | Hong | 2003 | 2009 | 1334 | 66.4 | - |
| 24 | Zheng | 2004 | 112 | 47 | 42.0 | - |
| 25 | Yao | 2005 | 108 | 72 | 66.7 | S-S speech disorders test and dysarthria test |
| 27 | Liao | 2005 | 55 | 38 | 69.1 | - |
| 30 | Li | 2006 | 126 | 48 | 38.1 | S-S speech disorders test and dysarthria test |
| 31 | Liu | 2006 | 118 | 47 | 39.8 | - |
| 34 | Zhang | 2007 | 80 | 53 | 66.3 | S-S speech disorders test and dysarthria test |
| 36 | Zhou | 2007 | 285 | 214 | 75.1 | - |
| 37 | Wang | 2007 | 484 | 194 | 40.1 | - |
| 38 | Huang | 2008 | 258 | 136 | 52.7 | - |
| 40 | Sun | 2008 | 81 | 50 | 61.7 | - |
| 44 | Zhou | 2009 | 680 | 89 | 13.1 | - |
| 46 | [Liu](https://www.webofscience.com/wos/author/record/28777125) | 2009 | 90 | 44 | 48.9 | - |
| 48 | Wang | 2010 | 290 | 152 | 52.4 | S-S speech disorders test and dysarthria test |
| 49 | Rui | 2010 | 394 | 22 | 5.6 | - |
| 52 | Hou | 2010 | 354 | 216 | 61.0 | - |
| 54 | Li | 2011 | 208 | 86 | 41.3 | - |
| 55 | Wang | 2011 | 1204 | 524 | 43.5 | - |
| 58 | [Qin](http://med.wanfangdata.com.cn/Author/General/A000035461) | 2011 | 1090 | 580 | 53.2 | - |
| 59 | Huang | 2012 | 273 | 144 | 52.7 | - |
| 61 | Zhou | 2012 | 530 | 119 | 22.5 | S-S speech disorders test and dysarthria test |
| 66 | Jia | 2014 | 182 | 117 | 64.3 | - |
| 69 | Wang | 2016 | 327 | 194 | 59.3 | S-S speech disorders test and dysarthria test |
| 73 | Guan | 2017 | 1323 | 531 | 40.1 | - |
| 78 | Ke | 2018 | 300 | 97 | 32.3 | - |
| 80 | Chiang | 2019 | 8419 | 1538 | 18.3 | - |
| 81 | Yuan | 2020 | 769 | 424 | 55.1 | S-S speech disorders test and dysarthria test |
| Total | - | - | 21426 | 7687 | - | - |

**Table 4. Summary of articles reporting hearing disorder related information**

| **No.** | **author** | **years** | **n** | **event** | **Prevalence (%)** | **Diagnostic criteria for hearing disorders** |
| --- | --- | --- | --- | --- | --- | --- |
| 16 | Liu | 2000 | 83 | 2 | 2.4 | - |
| 17 | Cao | 2001 | 136 | 43 | 31.6 | Brainstem auditory evoked potential (BAEP) |
| 19 | He | 2002 | 146 | 5 | 34.2 | - |
| 21 | Gao | 2003 | 582 | 69 | 11.9 | - |
| 22 | Hong | 2003 | 2009 | 227 | 11.3 | - |
| 23 | Wang | 2004 | 265 | 12 | 4.5 | - |
| 24 | Zheng | 2004 | 112 | 22 | 19.6 | - |
| 25 | Yao | 2005 | 108 | 12 | 11.1 | BAEP |
| 27 | Liao | 2005 | 55 | 6 | 10.9 | - |
| 28 | Chan | 2005 | 181 | 27 | 14.9 | - |
| 29 | Zheng | 2006 | 195 | 12 | 6.2 | BAEP |
| 30 | Li | 2006 | 126 | 59 | 46.8 | BAEP |
| 31 | Liu | 2006 | 118 | 42 | 35.6 | BAEP |
| 34 | Zhang | 2007 | 80 | 9 | 11.3 | BAEP |
| 35 | Liu | 2007 | 74 | 9 | 12.2 | BAEP |
| 37 | Wang | 2007 | 484 | 160 | 33.1 | BAEP |
| 38 | Huang | 2008 | 258 | 32 | 12.4 | BAEP |
| 40 | Sun | 2008 | 81 | 9 | 11.1 | - |
| 48 | Wang | 2010 | 290 | 88 | 30.3 | BAEP |
| 49 | Rui | 2010 | 394 | 10 | 2.5 | BAEP |
| 52 | Hou | 2010 | 354 | 15 | 4.2 | - |
| 54 | Li | 2011 | 208 | 4 | 1.9 | - |
| 55 | Wang | 2011 | 1204 | 266 | 22.1 | - |
| 56 | Tang | 2011 | 120 | 38 | 31.7 | BAEP |
| 59 | Huang | 2012 | 273 | 27 | 9.9 | - |
| 61 | Zhou | 2012 | 530 | 183 | 34.5 | - |
| 63 | Wu | 2012 | 196 | 4 | 2.0 | - |
| 66 | Jia | 2014 | 182 | 33 | 18.1 | - |
| 67 | Guo | 2014 | 444 | 52 | 11.7 | - |
| 68 | Li | 2015 | 238 | 24 | 10.1 | - |
| 69 | Wang | 2016 | 327 | 31 | 9.5 | BAEP |
| 70 | Lin | 2016 | 125 | 53 | 42.4 | - |
| 71 | Chen | 2016 | 524 | 241 | 46.0 | BAEP |
| 73 | Guan | 2017 | 1323 | 54 | 4.1 | - |
| 74 | Shu | 2017 | 100 | 34 | 34 | BAEP |
| 77 | He | 2017 | 407065 | 28423 | 7.0 | - |
| 78 | Ke | 2018 | 300 | 38 | 12.7 | - |
| 79 | Yang | 2018 | 112 | 40 | 35.7 | BAEP |
| 80 | Chiang | 2019 | 8419 | 142 | 1.7 | - |
| 81 | Yuan | 2020 | 1117 | 158 | 14.1 | BAEP |
| 86 | Zhu | 2022 | 94 | 25 | 26.6 | BAEP |
| Total | - | - | 429032 | 30740 | - | - |

**Table 5. Summary of articles reporting vision disorder related information**

| **No.** | **author** | **years** | **n** | **event** | **Prevalence (%)** | **Diagnostic criteria for vision disorders** |
| --- | --- | --- | --- | --- | --- | --- |
| 16 | Liu | 2000 | 83 | 2 | 2.4 | - |
| 21 | Gao | 2003 | 582 | 38 | 6.5 | - |
| 22 | Hong | 2003 | 2009 | 117 | 5.8 | - |
| 23 | Wang | 2004 | 262 | 15 | 5.7 | - |
| 24 | Zheng | 2004 | 112 | 16 | 14.3 | - |
| 25 | Yao | 2005 | 108 | 6 | 5.6 | Visual evoked potential(VEP) |
| 28 | Chan | 2005 | 181 | 114 | 63.0 | - |
| 29 | Zheng | 2006 | 195 | 92 | 47.2 | Ophthalmic examination |
| 30 | Li | 2006 | 126 | 32 | 25.4 | Ophthalmic examination |
| 31 | Liu | 2006 | 118 | 11 | 9.3 | - |
| 34 | Zhang | 2007 | 80 | 5 | 6.3 | VEP |
| 35 | Liu | 2007 | 74 | 17 | 23.0 | - |
| 36 | Zhou | 2007 | 285 | 52 | 18.2 | - |
| 38 | Huang | 2008 | 285 | 110 | 38.6 | - |
| 40 | Sun | 2008 | 81 | 6 | 7.4 | - |
| 42 | Liu | 2008 | 347 | 49 | 14.1 | Ophthalmic examination+VEP |
| 44 | Zhou | 2009 | 680 | 52 | 7.6 | - |
| 48 | Wang | 2010 | 290 | 77 | 26.6 | Ophthalmic examination |
| 49 | Rui | 2010 | 394 | 7 | 1.8 | - |
| 52 | Hou | 2010 | 354 | 138 | 39.0 | - |
| 54 | Li | 2011 | 208 | 91 | 43.8 | - |
| 59 | Huang | 2012 | 273 | 12 | 4.4 | - |
| 60 | Song | 2012 | 342 | 24 | 7.0 | - |
| 62 | Xiong | 2012 | 223 | 158 | 70.9 | Ophthalmic examination |
| 63 | Wu | 2012 | 196 | 101 | 51.5 | - |
| 66 | Jia | 2014 | 182 | 41 | 22.5 | - |
| 67 | Guo | 2014 | 444 | 55 | 12.4 | - |
| 68 | Li | 2015 | 238 | 33 | 13.9 | - |
| 69 | Wang | 2016 | 327 | 98 | 30.0 | Ophthalmic examination |
| 70 | Lin | 2016 | 125 | 53 | 42.4 | Ophthalmic examination |
| 72 | Li | 2016 | 265 | 127 | 47.9 | Ophthalmic examination |
| 73 | Guan | 2017 | 1323 | 70 | 5.3 | - |
| 77 | He | 2017 | 407065 | 20338 | 5.0 | - |
| 81 | Yuan | 2020 | 1117 | 109 | 9.8 | - |
| 85 | Lin | 2022 | 176 | 105 | 59.7 | Ophthalmic examination |
| Total | - | - | 419150 | 22371 | - | - |
