# Supplementary material for: Prevalence of cerebral palsy comorbidities in China: a systematic review and meta-analysis
Source: Front Neurol. 2023 Sep 28;14:1233700. doi: 10.3389/fneur.2023.1233700 (PMC10568468; doi:10.3389/fneur.2023.1233700)
Supplement: Supplementary file 3 [file Data_Sheet_3.docx]

| **Table Article quality evaluation by AHRQ** | | | | | | | | | | | | | | |
| --- | --- | --- | --- | --- | --- | --- | --- | --- | --- | --- | --- | --- | --- | --- |
| No. | First author | Years | 1 | 2 | 3 | 4 | 5 | 6 | 7 | 8 | 9 | 10 | 11 | Score |
| 14 | Yu | 1997 | Yes | No | Yes | Yes | Unclearly | No | No | No | No | Yes | No | 4 |
| 15 | Kwong | 1998 | Yes | Yes | Yes | Yes | Unclearly | Yes | No | Yes | No | Yes | No | 7 |
| 16 | Liu | 2000 | Yes | Yes | Yes | Yes | Unclearly | Yes | No | No | No | Yes | No | 6 |
| 17 | Cao | 2001 | Yes | No | Yes | No | Unclearly | No | No | No | No | Yes | No | 3 |
| 18 | Dong | 2002 | Yes | Yes | Yes | Yes | Unclearly | Yes | No | No | No | Yes | No | 6 |
| 19 | He | 2002 | Yes | No | Yes | Yes | Unclearly | No | No | No | No | Yes | No | 4 |
| 20 | Huang | 2002 | Yes | No | Yes | Yes | Unclearly | Yes | No | No | No | Yes | No | 5 |
| 21 | Gao | 2003 | Yes | No | Yes | No | Unclearly | Yes | Yes | Yes | No | Yes | No | 6 |
| 22 | Hong | 2003 | Yes | Yes | Yes | No | Unclearly | Yes | Yes | Yes | No | Yes | No | 7 |
| 23 | Wang | 2004 | Yes | No | Yes | No | Unclearly | Yes | No | No | No | Yes | No | 4 |
| 24 | Zheng | 2004 | Yes | No（Partial outcome not described） | Yes | No | Unclearly | No（Partial outcome not described） | No | No | No | Yes | No | 3 |
| 25 | Yao | 2005 | Yes | Yes | Yes | No | Unclearly | Yes | No | No | No | Yes | No | 5 |
| 26 | Lai | 2005 | Yes | Yes | Yes | Yes | Unclearly | Yes | No | No | No | Yes | No | 6 |
| 27 | Liao | 2005 | Yes | No | Yes | Yes | Unclearly | No | No | No | No | Yes | No | 4 |
| 28 | Chan | 2005 | Yes | No | Yes | No | Unclearly | No | No | No | No | Yes | No | 3 |
| 29 | Zheng | 2006 | Yes | No | Yes | No | Unclearly | Yes | No | No | No | Yes | No | 4 |
| 30 | Li | 2006 | Yes | No | Yes | No | Unclearly | Yes | No | No | No | Yes | No | 4 |
| 31 | Liu | 2006 | Yes | No | Yes | No | Unclearly | No（Partial outcome not described） | No | No | No | Yes | No | 3 |
| 32 | Wang | 2006 | Yes | No | Yes | No | Unclearly | Yes | No | No | No | Yes | No | 4 |
| 33 | Cao | 2007 | Yes | No | Yes | No | Unclearly | No | No | No | No | Yes | No | 3 |
| 34 | Zhang | 2007 | Yes | No | Yes | No | Unclearly | No（Partial outcome not described） | No | No | No | Yes | No | 3 |
| 35 | Liu | 2007 | Yes | No | Yes | No | Unclearly | No | No | No | No | Yes | No | 3 |
| 36 | Zhou | 2007 | Yes | No | Yes | Yes | Unclearly | No | No | No | No | Yes | No | 4 |
| 37 | Wang | 2007 | Yes | No（Partial outcome not described） | Yes | Yes | Unclearly | No（Partial outcome not described） | Yes | No | No | Yes | No | 5 |
| 38 | Huang | 2008 | Yes | No | Yes | No | Unclearly | No（Partial outcome not described） | No | No | No | Yes | No | 3 |
| 39 | Li | 2008 | Yes | Yes | Yes | Yes | Unclearly | Yes | No | No | No | Yes | No | 6 |
| 40 | Sun | 2008 | Yes | No | Yes | Yes | Unclearly | Yes | No | No | No | Yes | No | 5 |
| 41 | Hou | 2008 | Yes | No | Yes | No | Unclearly | No | No | No | No | Yes | No | 3 |
| 42 | Liu | 2008 | Yes | No | Yes | Yes | Unclearly | Yes | No | No | No | Yes | No | 5 |
| 43 | Zhang | 2009 | Yes | No | Yes | No | Unclearly | Yes | No | No | No | Yes | No | 4 |
| 44 | Zhou | 2009 | Yes | No | Yes | Yes | Unclearly | No | No | No | No | Yes | No | 4 |
| 45 | Li | 2009 | Yes | No | Yes | No | Unclearly | No（Partial outcome not described） | No | No | No | Yes | No | 3 |
| 46 | [Liu](https://www.webofscience.com/wos/author/record/28777125) | 2009 | Yes | No | No | No | Unclearly | Yes | No | No | No | Yes | No | 3 |
| 47 | Wen | 2010 | Yes | Yes | Yes | No | Unclearly | Yes | No | No | No | Yes | No | 5 |
| 48 | Wang | 2010 | Yes | No | Yes | Yes | Unclearly | Yes | No | No | No | Yes | No | 5 |
| 49 | Rui | 2010 | Yes | No | Yes | No | Unclearly | Yes | No | No | No | Yes | No | 4 |
| 50 | Chu | 2010 | Yes | Yes | Yes | No | Unclearly | Yes | No | No | No | Yes | No | 5 |
| 51 | Zhu | 2010 | Yes | Yes | Yes | Yes | Unclearly | Yes | Yes | No | No | Yes | No | 7 |
| 52 | Hou | 2010 | Yes | Yes | Yes | No | Unclearly | Yes | No | No | No | Yes | No | 5 |
| 53 | [Huang](https://www.webofscience.com/wos/author/record/1605637) | 2010 | Yes | Yes | Yes | No | Unclearly | Yes | Yes | Yes | No | Yes | No | 7 |
| 54 | Li | 2011 | Yes | No | Yes | Yes | Unclearly | No | No | No | No | Yes | No | 4 |
| 55 | Wang | 2011 | Yes | No | Yes | Yes | Unclearly | Yes | No | No | No | Yes | No | 5 |
| 56 | Tang | 2011 | Yes | Yes | Yes | No | Unclearly | Yes | No | No | No | Yes | No | 5 |
| 57 | Wu | 2011 | Yes | Yes | Yes | No | Unclearly | Yes | No | No | No | Yes | No | 5 |
| 58 | Qin | 2011 | Yes | No | Yes | No | Unclearly | No | No | No | No | Yes | No | 3 |
| 59 | Huang | 2012 | Yes | No | Yes | No | Unclearly | No | No | No | No | Yes | No | 3 |
| 60 | Song | 2012 | Yes | No | Yes | Yes | Unclearly | No | No | Yes | No | Yes | No | 5 |
| 61 | Zhou | 2012 | Yes | No | Yes | No | Unclearly | No | No | No | No | Yes | No | 3 |
| 62 | Xiong | 2012 | Yes | Yes | Yes | No | Unclearly | Yes | No | No | No | Yes | No | 5 |
| 63 | Wu | 2012 | Yes | No | No | No | Unclearly | No | Yes | No | No | Yes | No | 3 |
| 64 | Peng | 2013 | Yes | Yes | Yes | Yes | Unclearly | Yes | Yes | No | No | Yes | No | 7 |
| 65 | Sun | 2013 | Yes | Yes | Yes | No | Unclearly | Yes | No | No | No | Yes | No | 5 |
| 66 | Jia | 2014 | Yes | No | Yes | Yes | Unclearly | No | Yes | No | No | Yes | No | 5 |
| 67 | Guo | 2014 | Yes | Yes | Yes | Yes | Unclearly | No | No | No | No | Yes | No | 5 |
| 68 | Li | 2015 | Yes | No | Yes | Yes | Unclearly | Yes | No | No | No | Yes | No | 5 |
| 69 | Wang | 2016 | Yes | Yes | Yes | Yes | Unclearly | Yes | Yes | No | No | Yes | No | 7 |
| 70 | Lin | 2016 | Yes | No | Yes | Yes | Unclearly | Yes | Yes | No | No | Yes | No | 6 |
| 71 | Chen | 2016 | Yes | No | Yes | No | Unclearly | Yes | Yes | Yes | No | Yes | No | 6 |
| 72 | Li | 2016 | Yes | No | Yes | No | Unclearly | Yes | No | No | No | Yes | No | 4 |
| 73 | Guan | 2017 | Yes | No | Yes | No | Unclearly | No | Yes | Yes | No | Yes | No | 5 |
| 74 | Shu | 2017 | Yes | No | No | No | Unclearly | Yes | No | No | No | Yes | No | 3 |
| 75 | Xie | 2017 | Yes | Yes | Yes | No | Unclearly | Yes | Yes | Yes | No | Yes | No | 7 |
| 76 | Zhang | 2017 | Yes | Yes | Yes | No | Unclearly | Yes | Yes | No | No | Yes | No | 7 |
| 77 | He | 2017 | Yes | Yes | Yes | Yes | Unclearly | No | Yes | Yes | No | Yes | No | 7 |
| 78 | Ke | 2018 | Yes | No | Yes | No | Unclearly | No | No | Yes | No | Yes | No | 4 |
| 79 | Yang | 2018 | Yes | Yes | Yes | No | Unclearly | Yes | No | Yes | No | Yes | No | 6 |
| 80 | Chiang | 2019 | Yes | No | Yes | Yes | Unclearly | No | Yes | Yes | No | Yes | No | 6 |
| 81 | Yuan | 2020 | Yes | Yes | Yes | Yes | Unclearly | No | Yes | Yes | No | Yes | No | 7 |
| 82 | Wang | 2022 | Yes | Yes | Yes | Yes | Unclearly | Yes | Yes | No | No | Yes | No | 7 |
| 83 | Niu | 2022 | Yes | Yes | Yes | Yes | Unclearly | No | Yes | Yes | No | Yes | No | 7 |
| 84 | Yang | 2022 | Yes | No | Yes | No | Unclearly | No | Yes | No | No | Yes | No | 4 |
| 85 | Lin | 2022 | Yes | Yes | Yes | No | Unclearly | Yes | No | Yes | No | Yes | No | 6 |
| 86 | Zhu | 2022 | Yes | Yes | Yes | No | Unclearly | Yes | No | No | No | Yes | No | 5 |
| 1. Whether the data sources are identified (investigation, literature review);2. Whether inclusion and exclusion criteria for exposed and non-exposed groups (cases and controls) are listed, or reference is made to previous publications;3. Whether the period for identifying patients is given (during which patients are included in the study);4. If not from the population, whether the study subjects are continuous (whether all patients in a certain period of time are included in the study);5. Whether the evaluator of the subjective indicators of the patient is isolated from other objective indicators of the patient (for example, the evaluator cannot know the results of the patient's fracture site when assessing the degree of pain);6. Describes any assessment to ensure quality (e.g., testing/retesting of primary outcome indicators);7. The explanation is provided for the exclusion analysis of some patients;8. Measures to evaluate and control confounding factors are described;9. If missing values exist, explain how the data was processed in the analysis;10. The patient response rate and data collection integrity were summarized;11. If follow-up is available, the percentage of patients identified with expected incomplete data or follow-up results are | | | | | | | | | | | | | | |
