# Supplementary material for: Prevalence of cerebral palsy comorbidities in China: a systematic review and meta-analysis
Source: Front Neurol. 2023 Sep 28;14:1233700. doi: 10.3389/fneur.2023.1233700 (PMC10568468; doi:10.3389/fneur.2023.1233700)
Supplement: Supplementary file 4 [file Data_Sheet_4.docx]

**Table 1. Sensitivity analysis of total comorbidities**

| Remove article | | | Heterogeneity | | **95% (CI)** | | Prevalence **(%)** |
| --- | --- | --- | --- | --- | --- | --- | --- |
| **author** | **years** | **No.** | I^2^**(%)** | P value | low | high |  |
| **All articles** | | | **98.36** | **<0.001** | **73.8** | **85.7** | **79.7** |
| Dong | 2002 | 18 | 98.51 | <0.001 | 73.9 | 86.4 | 80.2 |
| He | 2002 | 19 | 97.39 | <0.001 | 77.1 | 86.6 | 81.9 |
| Gao | 2003 | 21 | 98.38 | <0.001 | 74.5 | 86.7 | 80.6 |
| Hong | 2003 | 22 | 98.23 | <0.001 | 73.4 | 86.2 | 79.8 |
| Yao | 2005 | 25 | 98.55 | <0.001 | 73.7 | 86.3 | 80.0 |
| Zheng | 2006 | 29 | 98.51 | <0.001 | 72.9 | 85.6 | 79.3 |
| Zhang | 2007 | 34 | 98.55 | <0.001 | 73.7 | 86.3 | 80.0 |
| Sun | 2008 | 40 | 98.55 | <0.001 | 73 | 85.7 | 79.3 |
| Wang | 2010 | 48 | 98.51 | <0.001 | 73.9 | 86.5 | 80.2 |
| Wang | 2011 | 55 | 97.78 | <0.001 | 72.7 | 85.0 | 78.9 |
| Huang | 2012 | 59 | 98.53 | <0.001 | 73.7 | 86.3 | 80.0 |
| Wu | 2012 | 63 | 98.56 | <0.001 | 73.4 | 86.1 | 79.7 |
| Jia | 2014 | 66 | 98.48 | <0.001 | 72.8 | 85.4 | 79.1 |
| Guo | 2014 | 67 | 96.22 | <0.001 | 72.8 | 84.0 | 78.4 |
| Li | 2015 | 68 | 98.54 | <0.001 | 73.1 | 85.9 | 79.5 |
| Wang | 2016 | 69 | 98.24 | <0.001 | 72.7 | 84.8 | 78.8 |

**Table 2. Sensitivity analysis of epilepsy**

| **Remove article** | | | **Heterogeneity** | | **95% (CI)** | | **Prevalence (%)** |
| --- | --- | --- | --- | --- | --- | --- | --- |
| **author** | **years** | **No.** | I^2^**(%)** | **P value** | **low** | **high** |  |
| **All articles** | | | **76.59** | **<0.001** | **15.4** | **20.4** | **17.9** |
| Yu | 1997 | 14 | 77.03 | <0.001 | 15.5 | 20.5 | 18 |
| Kwong | 1998 | 15 | 76.13 | <0.001 | 15.2 | 20.1 | 17.6 |
| Liu | 2000 | 16 | 76.99 | <0.001 | 15.5 | 20.5 | 18 |
| Cao | 2001 | 17 | 77.14 | <0.001 | 15.4 | 20.5 | 18 |
| Dong | 2002 | 18 | 77.22 | <0.001 | 15.4 | 20.4 | 17.9 |
| He | 2002 | 19 | 77.20 | <0.001 | 15.4 | 20.4 | 17.9 |
| Gao | 2003 | 21 | 77.10 | <0.001 | 15.4 | 20.5 | 17.9 |
| Hong | 2003 | 22 | 76.14 | <0.001 | 15.4 | 20.5 | 17.9 |
| Wang | 2004 | 23 | 76.65 | <0.001 | 15.6 | 20.6 | 18.1 |
| Zheng | 2004 | 24 | 77.14 | <0.001 | 15.3 | 20.4 | 17.9 |
| Yao | 2005 | 25 | 77.16 | <0.001 | 15.4 | 20.4 | 17.9 |
| Lai | 2005 | 26 | 76.96 | <0.001 | 15.3 | 20.3 | 17.8 |
| Liao | 2005 | 27 | 77.09 | <0.001 | 15.4 | 20.4 | 17.9 |
| Chan | 2005 | 28 | 76.17 | <0.001 | 15.2 | 20.1 | 17.6 |
| Zheng | 2006 | 29 | 77.11 | <0.001 | 15.5 | 20.5 | 18 |
| Li | 2006 | 30 | 77.18 | <0.001 | 15.4 | 20.4 | 17.9 |
| Wang | 2006 | 32 | 76.75 | <0.001 | 15.6 | 20.6 | 18.1 |
| Cao | 2007 | 33 | 77.12 | <0.001 | 15.3 | 20.4 | 17.9 |
| Zhang | 2007 | 34 | 77.13 | <0.001 | 15.4 | 20.4 | 17.9 |
| Liu | 2007 | 35 | 77.02 | <0.001 | 15.5 | 20.5 | 18 |
| Zhou | 2007 | 36 | 76.23 | <0.001 | 15.7 | 20.6 | 18.1 |
| Wang | 2007 | 37 | 76.67 | <0.001 | 15.6 | 20.6 | 18.1 |
| Huang | 2008 | 38 | 77.22 | <0.001 | 15.4 | 20.4 | 17.9 |
| Li | 2008 | 39 | 76.76 | <0.001 | 15.5 | 20.6 | 18.1 |
| Sun | 2008 | 40 | 76.84 | <0.001 | 15.5 | 20.5 | 18 |
| Hou | 2008 | 41 | 77.12 | <0.001 | 15.5 | 20.5 | 18 |
| Zhang | 2009 | 43 | 73.70 | <0.001 | 15.1 | 19.8 | 17.5 |
| Zhou | 2009 | 44 | 76.46 | <0.001 | 15.6 | 20.6 | 18.1 |
| Li | 2009 | 45 | 76.74 | <0.001 | 15.6 | 20.6 | 18.1 |
| [Liu](https://www.webofscience.com/wos/author/record/28777125) | 2009 | 46 | 77.13 | <0.001 | 15.3 | 20.4 | 17.9 |
| Wen | 2010 | 47 | 76.85 | <0.001 | 15.2 | 20.3 | 17.7 |
| Wang | 2010 | 48 | 77.20 | <0.001 | 15.3 | 20.4 | 17.9 |
| Rui | 2010 | 49 | 75.9 | <0.001 | 15.7 | 20.6 | 18.2 |
| Zhu | 2010 | 51 | 76.67 | <0.001 | 15.3 | 20.4 | 17.9 |
| Li | 2011 | 54 | 77.04 | <0.001 | 15.5 | 20.5 | 18 |
| Wang | 2011 | 55 | 76.43 | <0.001 | 15.2 | 20.3 | 17.8 |
| Wu | 2011 | 57 | 76.81 | <0.001 | 15.2 | 20.2 | 17.7 |
| [Qin](http://med.wanfangdata.com.cn/Author/General/A000035461) | 2011 | 58 | 74.82 | <0.001 | 15.8 | 20.7 | 18.2 |
| Huang | 2012 | 59 | 77.20 | <0.001 | 15.4 | 20.5 | 17.9 |
| Zhou | 2012 | 61 | 76.00 | <0.001 | 15.1 | 20.1 | 17.6 |
| Wu | 2012 | 63 | 75.90 | <0.001 | 15.1 | 20.1 | 17.6 |
| Peng | 2013 | 64 | 77.13 | <0.001 | 15.4 | 20.4 | 17.9 |
| Sun | 2013 | 65 | 76.97 | <0.001 | 15.3 | 20.3 | 17.8 |
| Jia | 2014 | 66 | 77.17 | <0.001 | 15.4 | 20.5 | 17.9 |
| Guo | 2014 | 67 | 76.65 | <0.001 | 15.6 | 20.6 | 18.1 |
| Li | 2015 | 68 | 73.68 | <0.001 | 15.1 | 19.8 | 17.4 |
| Wang | 2016 | 69 | 75.86 | <0.001 | 15.1 | 20.1 | 17.6 |
| Guan | 2017 | 73 | 75.81 | <0.001 | 15.6 | 20.6 | 18.1 |
| Xie | 2017 | 75 | 77.14 | <0.001 | 15.3 | 20.4 | 17.8 |
| Zhang | 2017 | 76 | 76.93 | <0.001 | 15.5 | 20.5 | 18 |
| Ke | 2018 | 78 | 77.18 | <0.001 | 15.4 | 20.5 | 17.9 |
| Chiang | 2019 | 80 | 69.47 | <0.001 | 15.1 | 20 | 17.5 |
| Yuan | 2020 | 81 | 76.51 | <0.001 | 15.4 | 20.5 | 18 |
| Wang | 2022 | 82 | 77.16 | <0.001 | 15.4 | 20.4 | 17.9 |
| Niu | 2022 | 83 | 76.60 | <0.001 | 15.2 | 20.3 | 17.7 |
| [Yang](http://qikan.cqvip.com/Qikan/Search/Index?key=A=%e6%9d%a8%e4%b9%90) | 2022 | 84 | 76.68 | <0.001 | 15.6 | 20.6 | 18.1 |

**Table 3. Sensitivity analysis of intellectual disability**

| **Remove article** | | | **Heterogeneity** | | **95% (CI)** | | **Prevalence (%)** |
| --- | --- | --- | --- | --- | --- | --- | --- |
| **author** | **years** | **No.** | I^2^**(%)** | **P value** | **low** | **high** |  |
| **All articles** | | | **99.11** | **<0.001** | **51.8** | **64.3** | **58.0** |
| Yu | 1997 | 14 | 99.13 | <0.001 | 51.4 | 64.1 | 57.8 |
| Liu | 2000 | 16 | 99.12 | <0.001 | 52.3 | 64.8 | 58.5 |
| Cao | 2001 | 17 | 99.13 | <0.001 | 51.3 | 63.9 | 57.6 |
| Dong | 2002 | 18 | 99.14 | <0.001 | 51.3 | 64.0 | 57.6 |
| He | 2002 | 19 | 99.12 | <0.001 | 52.2 | 64.7 | 58.5 |
| Gao | 2003 | 21 | 99.15 | <0.001 | 51.7 | 64.5 | 58.1 |
| Hong | 2003 | 22 | 99.08 | <0.001 | 51.4 | 64.2 | 57.8 |
| Wang | 2004 | 23 | 99.10 | <0.001 | 51.0 | 63.5 | 57.2 |
| Zheng | 2004 | 24 | 99.15 | <0.001 | 52.0 | 64.6 | 58.3 |
| Yao | 2005 | 25 | 99.16 | <0.001 | 51.4 | 64.2 | 57.8 |
| Liao | 2005 | 27 | 99.14 | <0.001 | 51.1 | 63.8 | 57.4 |
| Chan | 2005 | 28 | 99.16 | <0.001 | 51.4 | 64.2 | 57.8 |
| Zheng | 2006 | 29 | 99.11 | <0.001 | 51.0 | 63.5 | 57.3 |
| Li | 2006 | 30 | 99.16 | <0.001 | 51.5 | 64.3 | 57.9 |
| Liu | 2006 | 31 | 99.16 | <0.001 | 51.5 | 64.3 | 57.9 |
| Wang | 2006 | 32 | 99.15 | <0.001 | 51.3 | 64.1 | 57.7 |
| Zhang | 2007 | 34 | 99.16 | <0.001 | 51.4 | 64.2 | 57.8 |
| Liu | 2007 | 35 | 99.13 | <0.001 | 51.0 | 63.6 | 57.3 |
| Zhou | 2007 | 36 | 99.15 | <0.001 | 51.7 | 64.4 | 58.1 |
| Wang | 2007 | 37 | 99.15 | <0.001 | 51.5 | 64.3 | 57.9 |
| Huang | 2008 | 38 | 99.15 | <0.001 | 51.3 | 64.0 | 57.7 |
| Sun | 2008 | 40 | 99.16 | <0.001 | 51.4 | 64.1 | 57.7 |
| Hou | 2008 | 41 | 99.12 | <0.001 | 51.1 | 63.6 | 57.3 |
| Zhou | 2009 | 44 | 99.08 | <0.001 | 50.9 | 63.4 | 57.2 |
| Li | 2009 | 45 | 99.16 | <0.001 | 51.5 | 64.2 | 57.8 |
| Wang | 2010 | 48 | 99.15 | <0.001 | 51.4 | 64.2 | 57.8 |
| Rui | 2010 | 49 | 99.03 | <0.001 | 53.1 | 65.0 | 59.0 |
| Chu | 2010 | 50 | 99.14 | <0.001 | 51.1 | 63.7 | 57.4 |
| Huang | 2010 | 53 | 99.15 | <0.001 | 51.5 | 64.3 | 57.9 |
| Li | 2011 | 54 | 99.11 | <0.001 | 52.4 | 64.8 | 58.6 |
| Wang | 2011 | 55 | 99.09 | <0.001 | 51.2 | 63.9 | 57.6 |
| [Qin](http://med.wanfangdata.com.cn/Author/General/A000035461) | 2011 | 58 | 99.13 | <0.001 | 51.7 | 64.4 | 58.0 |
| Huang | 2012 | 59 | 99.16 | <0.001 | 51.7 | 64.4 | 58.0 |
| Zhou | 2012 | 61 | 99.10 | <0.001 | 52.5 | 64.9 | 58.7 |
| Wu | 2012 | 63 | 99.15 | <0.001 | 51.3 | 64.0 | 57.7 |
| Sun | 2013 | 65 | 99.16 | <0.001 | 51.6 | 64.3 | 57.9 |
| Jia | 2014 | 66 | 99.16 | <0.001 | 51.4 | 64.1 | 57.7 |
| Guo | 2014 | 67 | 99.02 | <0.001 | 50.9 | 63.3 | 57.1 |
| Li | 2015 | 68 | 99.15 | <0.001 | 51.3 | 64.1 | 57.7 |
| Wang | 2016 | 69 | 99.15 | <0.001 | 51.4 | 64.2 | 57.8 |
| Guan | 2017 | 73 | 99.12 | <0.001 | 51.7 | 64.4 | 58.1 |
| Xie | 2017 | 75 | 99.16 | <0.001 | 51.6 | 64.4 | 58.0 |
| Zhang | 2017 | 76 | 99.15 | <0.001 | 51.3 | 64.0 | 57.6 |
| He | 2017 | 77 | 98.26 | <0.001 | 51.2 | 64.0 | 57.6 |
| Ke | 2018 | 78 | 99.10 | <0.001 | 52.5 | 64.9 | 58.7 |
| Chiang | 2019 | 80 | 98.89 | <0.001 | 52.9 | 65.0 | 58.9 |
| Yuan | 2020 | 81 | 99.14 | <0.001 | 52.0 | 64.6 | 58.3 |
| [Yang](http://qikan.cqvip.com/Qikan/Search/Index?key=A=%e6%9d%a8%e4%b9%90) | 2022 | 84 | 99.03 | <0.001 | 53.1 | 65.0 | 59.0 |

**Table 4. Sensitivity analysis of speech disorders**

| **Remove article** | | | **Heterogeneity** | | **95% (CI)** | | **Prevalence (%)** |
| --- | --- | --- | --- | --- | --- | --- | --- |
| **author** | **years** | **No.** | I^2^**(%)** | **P value** | **low** | **high** |  |
| **All articles** | | | **97.14** | **<0.001** | **41.6** | **54.4** | **48.0** |
| Yu | 1997 | 14 | 97.02 | <0.001 | 40.8 | 53.4 | 47.1 |
| Cao | 2001 | 17 | 97.05 | <0.001 | 40.8 | 53.5 | 47.2 |
| Dong | 2002 | 18 | 97.27 | <0.001 | 41.6 | 54.8 | 48.2 |
| He | 2002 | 19 | 97.21 | <0.001 | 42.1 | 55.1 | 48.6 |
| Gao | 2003 | 21 | 97.19 | <0.001 | 41.9 | 55.0 | 48.4 |
| Hong | 2003 | 22 | 96.66 | <0.001 | 40.9 | 53.9 | 47.4 |
| Zheng | 2004 | 24 | 97.28 | <0.001 | 41.7 | 54.8 | 48.3 |
| Yao | 2005 | 25 | 97.19 | <0.001 | 41.0 | 53.9 | 47.4 |
| Liao | 2005 | 27 | 97.19 | <0.001 | 41.0 | 53.9 | 47.4 |
| Li | 2006 | 30 | 97.27 | <0.001 | 41.8 | 54.9 | 48.3 |
| Liu | 2006 | 31 | 97.28 | <0.001 | 41.7 | 54.8 | 48.3 |
| Zhang | 2007 | 34 | 97.21 | <0.001 | 41.0 | 53.9 | 47.5 |
| Zhou | 2007 | 36 | 96.98 | <0.001 | 40.8 | 53.4 | 47.1 |
| Wang | 2007 | 37 | 97.23 | <0.001 | 41.7 | 54.9 | 48.3 |
| Huang | 2008 | 38 | 97.27 | <0.001 | 41.3 | 54.5 | 47.9 |
| Sun | 2008 | 40 | 97.25 | <0.001 | 41.1 | 54.1 | 47.6 |
| Zhou | 2009 | 44 | 96.84 | <0.001 | 43.0 | 55.3 | 49.2 |
| [Liu](https://www.webofscience.com/wos/author/record/28777125) | 2009 | 46 | 97.30 | <0.001 | 41.4 | 54.6 | 48.0 |
| Wang | 2010 | 48 | 97.26 | <0.001 | 41.3 | 54.5 | 47.9 |
| Rui | 2010 | 49 | 96.70 | <0.001 | 43.4 | 55.4 | 49.4 |
| Hou | 2010 | 52 | 97.20 | <0.001 | 41.1 | 54.2 | 47.6 |
| Li | 2011 | 54 | 97.27 | <0.001 | 41.7 | 54.8 | 48.2 |
| Wang | 2011 | 55 | 97.13 | <0.001 | 41.6 | 54.8 | 48.2 |
| [Qin](http://med.wanfangdata.com.cn/Author/General/A000035461) | 2011 | 58 | 97.11 | <0.001 | 41.3 | 54.4 | 47.8 |
| Huang | 2012 | 59 | 97.26 | <0.001 | 41.3 | 54.5 | 47.9 |
| Zhou | 2012 | 61 | 97.06 | <0.001 | 42.5 | 55.2 | 48.9 |
| Jia | 2014 | 66 | 97.20 | <0.001 | 41.0 | 54.0 | 47.5 |
| Wang | 2016 | 69 | 97.21 | <0.001 | 41.1 | 54.2 | 47.6 |
| Guan | 2017 | 73 | 97.11 | <0.001 | 41.7 | 54.9 | 48.3 |
| Ke | 2018 | 78 | 97.21 | <0.001 | 42.0 | 55.0 | 48.5 |
| Chiang | 2019 | 80 | 96.10 | <0.001 | 42.8 | 55.3 | 49.1 |
| Yuan | 2020 | 81 | 97.15 | <0.001 | 41.2 | 54.4 | 47.8 |

**Table 5. Sensitivity analysis of hearing disorders**

| **Remove article** | | | **Heterogeneity** | | **95% (CI)** | | **Prevalence (%)** |
| --- | --- | --- | --- | --- | --- | --- | --- |
| **author** | **years** | **No.** | I^2^**(%)** | **P value** | **low** | **high** |  |
| **All articles** | | | **94.75** | **<0.001** | **13.0** | **21.4** | **17.2** |
| Liu | 2000 | 16 | 94.88 | <0.001 | 13.2 | 21.7 | 17.5 |
| Cao | 2001 | 17 | 94.83 | <0.001 | 12.6 | 21.1 | 16.9 |
| He | 2002 | 19 | 94.86 | <0.001 | 13.3 | 21.8 | 17.5 |
| Gao | 2003 | 21 | 94.86 | <0.001 | 13.3 | 21.8 | 17.5 |
| Hong | 2003 | 22 | 94.53 | <0.001 | 13.3 | 21.8 | 17.5 |
| Wang | 2004 | 23 | 94.81 | <0.001 | 13.5 | 21.9 | 17.7 |
| Zheng | 2004 | 24 | 94.94 | <0.001 | 13.1 | 21.5 | 17.3 |
| Yao | 2005 | 25 | 94.93 | <0.001 | 13.3 | 21.7 | 17.5 |
| Liao | 2005 | 27 | 94.92 | <0.001 | 13.2 | 21.7 | 17.5 |
| Chan | 2005 | 28 | 94.95 | <0.001 | 13.2 | 21.7 | 17.4 |
| Zheng | 2006 | 29 | 94.86 | <0.001 | 13.4 | 21.8 | 17.6 |
| Li | 2006 | 30 | 94.20 | <0.001 | 12.6 | 20.6 | 16.6 |
| Liu | 2006 | 31 | 94.72 | <0.001 | 12.8 | 21.1 | 16.9 |
| Zhang | 2007 | 34 | 94.92 | <0.001 | 13.2 | 21.7 | 17.5 |
| Liu | 2007 | 35 | 94.93 | <0.001 | 13.2 | 21.7 | 17.4 |
| Wang | 2007 | 37 | 94.6 | <0.001 | 12.7 | 21.1 | 16.9 |
| Huang | 2008 | 38 | 94.92 | <0.001 | 13.2 | 21.7 | 17.5 |
| Sun | 2008 | 40 | 94.92 | <0.001 | 13.3 | 21.7 | 17.5 |
| Wang | 2010 | 48 | 94.77 | <0.001 | 12.8 | 21.2 | 17.0 |
| Rui | 2010 | 49 | 94.72 | <0.001 | 13.6 | 21.9 | 17.8 |
| Hou | 2010 | 52 | 94.78 | <0.001 | 13.5 | 21.9 | 17.7 |
| Li | 2011 | 54 | 94.78 | <0.001 | 13.5 | 21.9 | 17.7 |
| Wang | 2011 | 55 | 94.70 | <0.001 | 12.9 | 21.5 | 17.2 |
| Tang | 2011 | 56 | 94.81 | <0.001 | 12.8 | 21.2 | 17.0 |
| Huang | 2012 | 59 | 94.90 | <0.001 | 13.3 | 21.8 | 17.6 |
| Zhou | 2012 | 61 | 94.52 | <0.001 | 12.7 | 21.0 | 16.8 |
| Wu | 2012 | 63 | 94.78 | <0.001 | 13.5 | 21.9 | 17.7 |
| Jia | 2014 | 66 | 94.95 | <0.001 | 13.1 | 21.6 | 17.3 |
| Guo | 2014 | 67 | 94.89 | <0.001 | 13.3 | 21.8 | 17.5 |
| Li | 2015 | 68 | 94.91 | <0.001 | 13.3 | 21.8 | 17.5 |
| Wang | 2016 | 69 | 94.88 | <0.001 | 13.3 | 21.8 | 17.6 |
| Lin | 2016 | 70 | 94.45 | <0.001 | 12.7 | 20.8 | 16.7 |
| Chen | 2016 | 71 | 93.65 | <0.001 | 12.5 | 20.4 | 16.5 |
| Guan | 2017 | 73 | 94.55 | <0.001 | 13.6 | 22.0 | 17.8 |
| Shu | 2017 | 74 | 94.78 | <0.001 | 12.8 | 21.2 | 17.0 |
| He | 2017 | 77 | 89.23 | <0.001 | 13.5 | 21.9 | 17.7 |
| Ke | 2018 | 78 | 94.92 | <0.001 | 13.2 | 21.7 | 17.5 |
| Yang | 2018 | 79 | 94.72 | <0.001 | 12.8 | 21.1 | 16.9 |
| Chiang | 2019 | 80 | 92.34 | <0.001 | 13.7 | 22.0 | 17.9 |
| Yuan | 2020 | 81 | 94.76 | <0.001 | 13.2 | 21.7 | 17.5 |
| Zhu | 2022 | 86 | 94.90 | <0.001 | 12.9 | 21.4 | 17.2 |

**Table 6. Sensitivity analysis of vision disorders**

| **Remove article** | | | **Heterogeneity** | | **95% (CI)** | | **Prevalence (%)** |
| --- | --- | --- | --- | --- | --- | --- | --- |
| **author** | **years** | **No.** | **I^2^(%)** | **P value** | **low** | **high** |  |
| **All articles** | | | **97.13** | **<0.001** | **16.3** | **29.8** | **23.1** |
| Liu | 2000 | 16 | 97.2 | <0.001 | 16.8 | 30.4 | 23.6 |
| Gao | 2003 | 21 | 97.12 | <0.001 | 16.7 | 30.4 | 23.6 |
| Hong | 2003 | 22 | 96.81 | <0.001 | 16.8 | 30.5 | 23.6 |
| Wang | 2004 | 23 | 97.18 | <0.001 | 16.7 | 30.4 | 23.6 |
| Zheng | 2004 | 24 | 97.25 | <0.001 | 16.4 | 30.2 | 23.3 |
| Yao | 2005 | 25 | 97.21 | <0.001 | 16.7 | 30.4 | 23.5 |
| Chan | 2005 | 28 | 96.81 | <0.001 | 15.4 | 28.4 | 21.9 |
| Zheng | 2006 | 29 | 97.11 | <0.001 | 15.6 | 29.1 | 22.3 |
| Li | 2006 | 30 | 97.26 | <0.001 | 16.1 | 29.9 | 23.0 |
| Liu | 2006 | 31 | 97.23 | <0.001 | 16.6 | 30.3 | 23.4 |
| Zhang | 2007 | 34 | 97.22 | <0.001 | 16.6 | 30.3 | 23.5 |
| Liu | 2007 | 35 | 97.27 | <0.001 | 16.2 | 30.0 | 23.1 |
| Zhou | 2007 | 36 | 97.23 | <0.001 | 16.3 | 30.1 | 23.2 |
| Huang | 2008 | 38 | 97.17 | <0.001 | 15.7 | 29.5 | 22.6 |
| Sun | 2008 | 40 | 97.23 | <0.001 | 16.6 | 30.3 | 23.4 |
| Liu | 2008 | 42 | 97.21 | <0.001 | 16.4 | 30.2 | 23.3 |
| Zhou | 2009 | 44 | 97.11 | <0.001 | 16.7 | 30.4 | 23.5 |
| Wang | 2010 | 48 | 97.23 | <0.001 | 16.0 | 29.9 | 23.0 |
| Rui | 2010 | 49 | 97.12 | <0.001 | 16.9 | 30.5 | 23.7 |
| Hou | 2010 | 52 | 97.14 | <0.001 | 15.7 | 29.4 | 22.6 |
| Li | 2011 | 54 | 97.14 | <0.001 | 15.6 | 29.3 | 22.4 |
| Huang | 2012 | 59 | 97.17 | <0.001 | 16.8 | 30.5 | 23.6 |
| Song | 2012 | 60 | 97.17 | <0.001 | 16.7 | 30.4 | 23.5 |
| Xiong | 2012 | 62 | 96.50 | <0.001 | 15.3 | 27.9 | 21.6 |
| Wu | 2012 | 63 | 97.04 | <0.001 | 15.5 | 28.9 | 22.2 |
| Jia | 2014 | 66 | 97.25 | <0.001 | 16.2 | 30.0 | 23.1 |
| Guo | 2014 | 67 | 97.18 | <0.001 | 16.5 | 30.3 | 23.4 |
| Li | 2015 | 68 | 97.23 | <0.001 | 16.4 | 30.2 | 23.3 |
| Wang | 2016 | 69 | 97.21 | <0.001 | 15.9 | 29.8 | 22.8 |
| Lin | 2016 | 70 | 97.18 | <0.001 | 15.7 | 29.3 | 22.5 |
| Li | 2016 | 72 | 97.07 | <0.001 | 15.5 | 29.1 | 22.3 |
| Guan | 2017 | 73 | 96.96 | <0.001 | 16.8 | 30.5 | 23.6 |
| He | 2017 | 77 | 94.40 | <0.001 | 16.8 | 30.5 | 23.7 |
| Yuan | 2020 | 81 | 97.03 | <0.001 | 16.6 | 30.4 | 23.5 |
| Lin | 2022 | 85 | 96.90 | <0.001 | 15.4 | 28.6 | 22.0 |
